# Preclinical studies of RA475, a guanidine-substituted spirocyclic candidate RPN13/ADRM1 inhibitor for treatment of ovarian cancer

**DOI:** 10.1371/journal.pone.0305710

**Published:** 2024-07-11

**Authors:** Ravi K. Anchoori, Ssu-Hsueh Tseng, Hua-Ling Tsai, Vikrant Palande, Michelle A. Rudek, Richard B. S. Roden

**Affiliations:** 1 Department of Oncology, Johns Hopkins University, Baltimore, Maryland, United States of America; 2 Department of Pathology, Johns Hopkins University, Baltimore, Maryland, United States of America; 3 Department of Medicine, Johns Hopkins University, Baltimore, Maryland, United States of America; IHRC, Inc. (Human Resource Service Administration), UNITED STATES OF AMERICA

## Abstract

There is an urgent unmet need for more targeted and effective treatments for advanced epithelial ovarian cancer (EOC). The emergence of drug resistance is a particular challenge, but small molecule covalent inhibitors have promise for difficult targets and appear less prone to resistance. Michael acceptors are covalent inhibitors that form bonds with cysteines or other nucleophilic residues in the target protein. However, many are categorized as pan-assay interference compounds (PAINS) and considered unsuitable as drugs due to their tendency to react non-specifically. Targeting RPN13/ADRM1-mediated substrate recognition and deubiquitination by the proteasome 19S Regulatory Particle (RP) is a promising treatment strategy. Early candidate RPN13 inhibitors (iRPN13) produced a toxic accumulation of very high molecular weight polyubiquitinated substrates, resulting in therapeutic activity in mice bearing liquid or solid tumor models, including ovarian cancer; however, they were not drug-like (PAINS) because of their central piperidone core. Up284 instead has a central spiro-carbon ring. We hypothesized that adding a guanidine moiety to the central ring nitrogen of Up284 would produce a compound, RA475, with improved drug-like properties and therapeutic activity in murine models of ovarian cancer. RA475 produced a rapid accumulation of high molecular polyubiquitinated proteins in cancer cell lines associated with apoptosis, similar to Up284 although it was 3-fold less cytotoxic. RA475 competed binding of biotinylated Up284 to RPN13. RA475 shows improved solubility and distinct pharmacodynamic properties compared to Up284. Specifically, tetraubiquitin firefly luciferase expressed in leg muscle was stabilized in mice more effectively upon IP treatment with RA475 than with Up284. However, pharmacologic analysis showed that RA475 was more rapidly cleared from the circulation, and less orally available than Up284. RA475 shows reduced ability to cross the blood-brain barrier and *in vitro* inhibition of HERG. Treatment of mice with RA475 profoundly inhibited the intraperitoneal growth of the ID8-luciferase ovarian tumor model. Likewise, RA475 treatment of immunocompetent mice inhibited the growth of spontaneous genetically-engineered peritoneal tumor, as did weekly cisplatin dosing. The combination of RA475 and cisplatin significantly extended survival compared to individual treatments, consistent with synergistic cytotoxicity in vitro. In sum, RA475 is a promising candidate covalent RPN13i with potential utility for treatment of patients with advanced EOC in combination with cisplatin.

## Introduction

Ovarian cancer remains the most deadly and second most common gynecological cancer, with an estimated 19,880 cases and 12,810 deaths per annum in the US [1]. Treatment typically involves aggressive primary debulking surgery, and platinum-based chemotherapy; carboplatin plus taxane is standard, but heated intraperitoneal chemotherapy (HIPEC) with cisplatin is also used [2]. Angiogenesis inhibitor bevacizumab [3], poly ADP-ribose polymerase (PARP) inhibitors [4], folate receptor alpha (FRα) antibody conjugate, are also used [5]. Nevertheless, >80% of patients will relapse within 2 years and require multiple lines of therapy, comprising additional cycles of toxic platinum-based chemotherapy or second line treatments such as doxorubicin [6]. Patients who are platinum refractory/resistant following front-line treatment have the poorest prognosis, and with each additional line of therapy patients develop more drug resistant disease [7].

**Fig 1:**
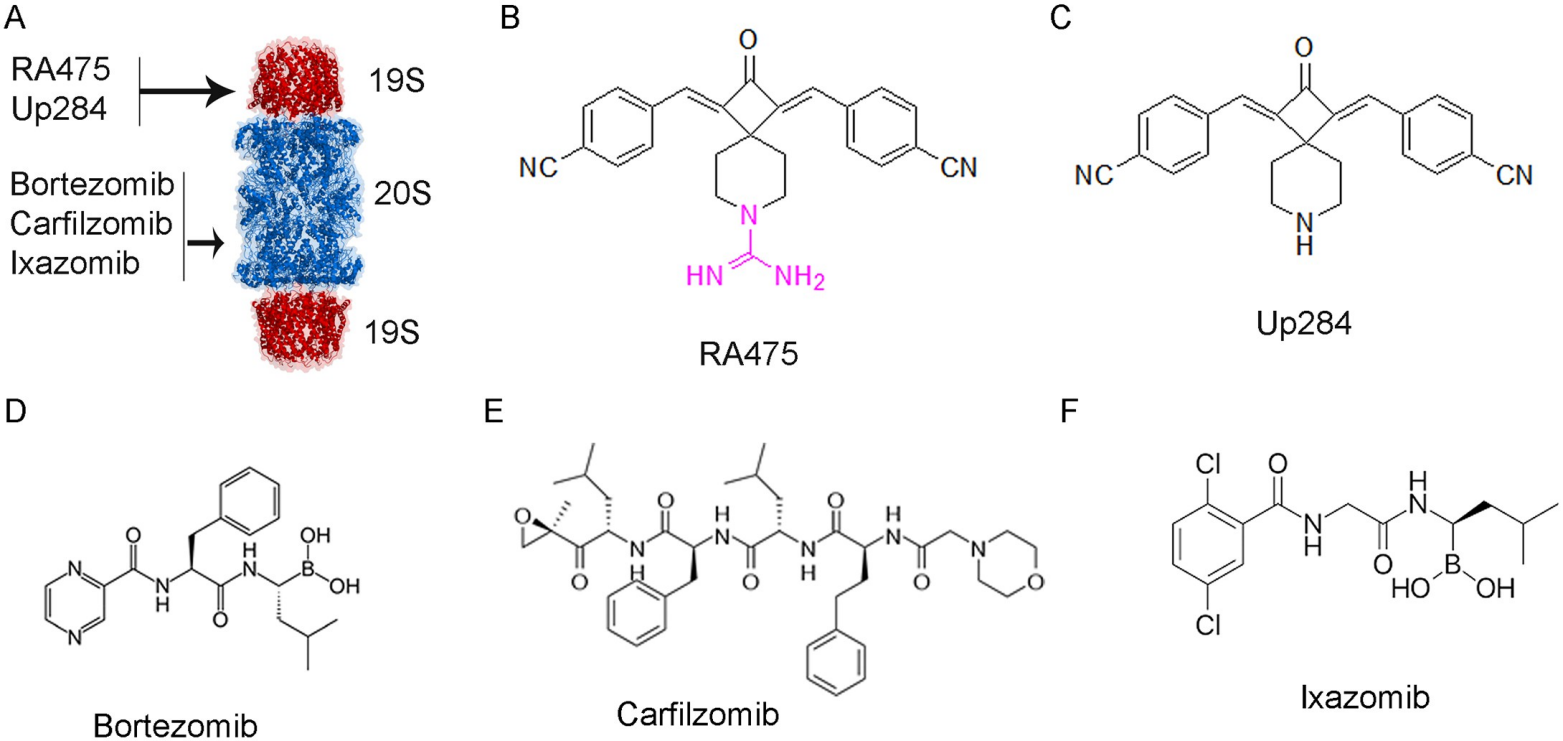
Structures. **A)** Drugs targeting proteasome components. **B)** Chemical structure of RA475. **C)** Chemical Structure of Up284, and of the licensed 20S proteasome inhibitors **D)** bortezomib, **E)** carfilzomib and **F)** ixazomib.

The success of inhibitors targeting proteasome 20S Catalytic Particle (CP) proteolytic activities in treating multiple myeloma, and promising *in vitro* data led to much interest in their use in treating solid tumors including ovarian cancer [8–10]. However, 20S proteasome inhibitors like bortezomib (Fig 1), even in combination with standard agents, were not sufficiently promising in early phase studies to develop further [11–14]. Intra peritoneal administration of bortezomib appeared more promising against ovarian cancer [15] than subcutaneous [16] or intra venous routes [17], suggesting access to tumor tissue may be a barrier to efficacy. In addition, the licensed 20S proteasome inhibitors have significant side effects (peripheral neuropathy, diarrhea, thrombocytopenia, neutropenia, anemia, hypotension, dyspnea etc. [18]) and resistance eventually emerges even in multiple myeloma [8]. Therefore, there is much interest in new chemical entities targeting the proteasome with fewer side effects and better penetration of solid tumors.

One new approach is to target the constitutive proteasome’s 19S Regulatory Particle (RP), for example with dienones [19]. VLX1570 adducts active site cysteine residues of two proteasome-associated deubiquitinases (DUB) USP14 and UCHL5 (also known as UCH37) [20, 21]. Deubiquitination in the 19S RP is a critical step in proteasomal degradation, and thus VLX1570 produces a toxic accumulation of polyubiquitinated substrates of molecular weights higher than seen with bortezomib and more rapidly [22, 23].

UCHL5/UCH37 DUB activity in the 19S RP is enhanced upon recognition of polyubiquitinated substrate by the 19S RP ubiquitin receptor RPN13 (also known as ADRM1, hRPN13, or GP110). RPN13 has an active site cysteine (C88) that is reactive with dienones like RA190 [24] and its derivatives [25–27]. These candidate small molecular inhibitors of RPN13 (iRPN13) also rapidly produce toxic accumulations of high molecular weight (HMW) polyubiquitinated substrates as with VLX1570, and exhibit therapeutic activity in mice bearing liquid [28] or solid tumor models [29, 30], including of ovarian cancer [24]. Further, they could overcome resistance to bortezomib [31].

As early iRPN13 candidates were not drug-like because of their central piperidone ring, a PAINS feature [32, 33], several groups have sought more drug-like iRPN13 [34, 35]. One recent approach was to replace the central piperidone ring with a more constrained 3,5 spirocyclic core [36]. Addition of nitrile electron withdrawing groups on both side rings produced the most effective 3,5 spirocyclic candidate, Up284 [37]. Here we sought to improve key drug-like properties of this molecule by adding a guanidine moiety to its central ring nitrogen, and examine its impact on the pharmaco-kinetic/dynamic profile of this new compound, RA475, and its therapeutic activity in murine models of ovarian cancer (Fig 1).

## Materials and methods

### Cell lines and cytotoxicity assays

All cell lines were obtained from the American Type Culture Collection (ATCC) and cultured in the specified medium supplemented with 10% fetal bovine serum, 100 IU/mL penicillin, and 100 μg/mL streptomycin at 37°C in a humidified 5% CO_2_/95% air incubator. Synthesis of new compounds is described in S1 File, and >95% purity of RA475, Up284 and its analogs were confirmed by NMR and MS. To assess drug cytotoxicity, cells were seeded at 2,500 cells/well (10,000 cells/well for MM lines) in 100 μL medium in 96-well plate as triplicates and after 24 h treated with compounds for a further 72 h. To assess cytotoxicity, the cells were incubated for 2 h at 37°C according to the manufacturer’s protocol with the Thiazolyl Blue Tetrazolium Bromide (20 μL/well of 5 mg/mL stock solution in water) (Sigma, M5655) and A_570_ measured using a Benchmark Plus microplate spectrophotometer (BIO-RAD). IC_50_ was estimated using Graphpad Prism 7 software.

### Antibodies and western blot analyses

Cell lysate prepared in MPER (Pierce) from each sample was normalized by total protein concentration using the bicinchoninic acid assay and bovine serum albumin standards. Normalized samples were subjected to SDS-PAGE, transferred to PVDF membranes and analyzed by Western blot using antibodies specific to ubiquitin (P4D1, sc-8017, Santa Cruz), PARP (#9542, BD Pharmingen), actin (#66009, Protein Tech Group), Tubulin(#66031, Protein Tech Group), ADRM1/RPN13 (D9Z1U, #12019, Cell Signaling), USP14 (Cell Signal, # 8159), Annexin V (#559763, BD Pharmingen), and for secondary antibodies we utilized either horseradish peroxidase (HRP)-linked anti-mouse IgG or anti-rabbit IgG (GE Healthcare UK Ltd), HRP-linked streptavidin (N100, Thermo Fisher) at the recommended dilution (1:5000).

### 4UbFL reporter assays

Sub-confluent cultures of cells were transfected with 4UbFL plasmid using TransIT 2020 reagent (Mirus Bio). Cells were seeded at 10,000 cells/well in 96-well plates 48 h post transfection, and incubated with compounds or vehicle (DMSO) at the doses and for the times indicated. Luciferase activity in cell lysate was determined with a luciferase assay kit (Promega) according to the manufacturer’s instructions. Bioluminescence was measured using a Glomax Multidetection system (Promega).

### Annexin V assay

The percentage of cells initiating apoptosis was assessed with the annexin V FITC assay kit. The cells (2 × 10^5^ cells/well) were treated with the described compounds at selected concentrations for 12 h or 16 h, as indicated. The cells were then collected, washed with PBS, resuspended in 100 μL of annexin V-FITC and 7-AAD dual-stain solution (3 μL Annexin V and 3 μL 7AAD, Catalog Number; 559763-BD bioscience), and incubated 20 min in the dark. The samples were then analyzed via flow cytometry using FlowJo V10 software. All experiments were repeated three times.

### Biotin labeling assay

Clarified cell lysate in MPER buffer (Pierce) was pretreated with streptavidin-coated magnetic beads for 45 min at 4°C to deplete non-specific biotinylated proteins in the cell lysate. The beads were separated and 40 μL of the pre-cleared cell lysate was incubated with compounds (20 μM) for 45 min at 4°C, and then treated with biotinylated compounds (10 μM) for 45 min at 4°C. Next, the samples were mixed with Laemmli sample buffer (BioRad) and boiled for 5 min. The proteins were separated using a 4–15% Bio-Rad Mini-PROTEAN SDS-PAGE gel (1 h at 100 V), and transferred to PVDF membrane overnight at 4°C (24 V). The membrane was blocked with 5% (w/v) BSA in phosphate buffered saline containing 0.1% (v/v) Tween 20 (PBST) for 1 h at RT, and washed for 20 min (3X with PBST). Then the membrane was probed with HRP-streptavidin (1:10,000 in PBST) for 1 h at RT, washed for 30 min (3X with PBST), and developed using HyGLO chemiluminescent detection reagent (Denville) and visualized using a Biorad Chemidoc imager.

### Microsomal, plasma and neat solution stability

Human and mouse liver microsomes and NADPH regenerating system solutions were purchased from Corning Life Sciences (Tewsbury, MA). Human and mouse K_2_EDTA-treated plasma was purchased from BioIVT (Westbury, NY). Metabolic studies in liver microsomes (0.125 mg/mL) were conducted in 1x PBS (pH 7.4), NADPH-generating system, and 10 μM RA475 in a final volume of 250 μL. An incubation mixture with or without NADPH-generating system, as well as a neat solution prepared without microsomes and NADPH-generating system was used. RA475 was added at the same concentration level into human and mouse EDTA plasma. All reactions were initiated with the addition of RA475. Incubations were performed in glass tubes maintained at 37°C in a shaker bath. At 0, 30, and 60 min, 10 μL samples were withdrawn from the reaction mixture, and 0.5 mL of acetonitrile containing internal standard was added to each. Samples were vortex-mixed and subjected to centrifugation for 5 min at 1430 *x g*. A 10 μL aliquot of the supernatant was injected onto the LC-MS/MS instrument for qualitative analysis using a temperature-controlled autosampling device operating at 5°C (See Pharmacokinetic section).

### Plasma Protein Binding (PPB)

The assay was performed in a multiple-use 96-well dialysis unit (HTD96b dialyzer). Each individual well unit consisted of 2 chambers separated by a vertically aligned dialysis membrane of predetermined pore size (MWCO 12–14 kDa). 120 μL of non-diluted plasma spiked with the compound (1 μM, final DMSO concentration 1%) was added to one chamber, and the same volume of PBS buffer pH 7.4 to the other chamber. The HTD96b dialyzer was covered with adhesive sealing film and incubated 5 h at 37°C while shaking at 100 rpm. For sample preparation, an aliquot of the contents of each chamber was mixed with the same volume of the blank opposite matrix. In order to define non-specific loss of the compound during this assay, a standard solution was created by mixing an aliquot of spiked plasma with blank buffer without dialysis. Two aliquots of the standard solution were incubated at 37°C, shaking at 100 rpm for 5 h (recovery samples). The other two aliquots were immediately diluted with acetonitrile and stored at 4°C until analysis (stability samples). All samples were diluted 5-fold with internal standard diluted in 100% methanol with subsequent plasma proteins sedimentation by centrifuging at 6000 rpm for 5 minutes. Supernatants were analyzed using LC-MS/MS. The percentage of plasma protein bound compound, recovery and stability were calculated using following equations.


$$\text{Protein}\;\text{binding}=\left\{\left(1-(\text{area}\;\text{ratio}\;\text{in}\;\text{buffer})/(\text{area}\;\text{ratio}\;\text{in}\;\text{plasma}\right)\right\}.100\%$$

$$\text{Recovery}=\left\{(\text{area}\;\text{ratio}\;\text{in}\;\text{buffer}\;+\;\text{peak}\;\text{area}\;\text{in}\;\text{plasma})/\text{area}\;\text{ratio}\;\text{in}\;\text{recovery}\;\text{sample}\right\}.100\%$$

$$\text{Stability}=\left\{\text{area}\;\text{ratio}\;\text{in}\;\text{recovery}\;\text{sample}/\text{area}\;\text{ratio}\;\text{in}\;\text{stability}\;\text{sample}\right\}.100\%$$

### LogD measurement

Incubations were carried out in Eppendorf-type polypropylene microtubes in triplicates. A 2.5 μL aliquot of 20 mM DMSO stock of a test compound was added into the previously mutually saturated mixture containing 500 μL of PBS (pH 7.4) and 500 μL of octanol. The solution was allowed to mix in a rotator for 1 hour at 30 rpm. Phase separation was assured by centrifugation for 2 min at 6000 rpm. The octanol phase was diluted 100-fold with 40% acetonitrile, and the aqueous phase (PBS buffer) was diluted 10-fold. The samples (both phases) were analyzed using an HPLC system coupled with a tandem mass spectrometer. Mebendazole was used as a reference compound. Calculations of the partition ratios were carried out using the equation below.

$$D=\left(do.So\right)/\left(dp.Sp\right)$$

where:

*So–*peak area of the analyte in octanol phase

*SP–*peak area of the analyte in PBS buffer

*do–*dilution coefficient for octanol phase

*dp–*dilution coefficient for aqueous phase

### GSH reactivity assay

Test compounds (20 μM, final organic solvent concentration 10%) were incubated with 1 mM GSH in phosphate buffer (100 mM), pH 7.4 at 37°C, shaking at 300 rpm. In the control reactions (“without GSH”), the GSH was substituted with phosphate buffer. Five time points over 1.5 h were analyzed (0, 0.33, 0.66, 1 and 1.5 h). The reactions were stopped by adding 50 μL aliquots of the reaction mixture to 9 volumes of methanol: water: formic acid (10:10:0.04) with internal standard (IS). Samples were analyzed using HPLC system coupled with a tandem mass spectrometer. Average (triplicate) area ratios were calculated. GSH reactivity was calculated taking into account the possible instability of the compound during incubation. All calculations were performed using normalized data obtained by dividing the average area ratio of the test compound incubated with GSH by the average area ratio of the test compound incubated without GSH. Natural logarithms of the results were fitted to linear regression, and half-life (t_1/2_) was calculated as:

$$t\frac{1}{2}=ln2/-slope$$

### Analysis of aqueous solubility

Kinetic solubility assays were performed according to Enamine’s aqueous solubility SOP. Briefly, using a 20 mM stock solution of the compound in 100% DMSO dilutions were prepared to a theoretical concentration of 400 μM in duplicates in phosphate-buffered saline pH 7.4 (138 mM NaCl, 2.7 mM KCl, 10 mM K-phosphate) with 2% final DMSO. The experimental compound dilutions in PBS were further allowed to equilibrate at 25°C on a thermostatic shaker for two hours and then filtered through HTS filter plates using a vacuum manifold. The filtrates of test compounds were diluted 2-fold with acetonitrile with 2% DMSO before measuring. In parallel, using a 20 mM stock solution of the compound in 100% DMSO dilutions were prepared to theoretical concentrations of 0 μM (blank), 10 μM, 25 μM, 50 μM, 100 μM, and 200 μM in 50% acetonitrile/PBS with 2% final DMSO to generate calibration curves. Ondansetron was used as a reference compound to control proper assay performance. 200 μL of each sample was transferred to a 96-well plate and measured in the 230–550 nm range with a 5 nm step. The concentrations of compounds in PBS filtrate are calculated using a dedicated Microsoft Excel calculation script. Proper absorbance wavelengths for calculations are selected for each compound manually based on absorbance maximums (absolute absorbance unit values for the minimum and maximum concentration points within the 0–3 OD range). Each final dataset is visually evaluated by the operator, and goodness of fit (R^2^) is calculated for each calibration curve. The effective range of this assay is approximately 2–400 μM and the compounds returning values close to the upper limit of the range may have higher actual solubility (e.g. 5’-deoxy-5-fluorouridine).

### Animal studies

All animal procedures were performed according to protocols approved by the Johns Hopkins University Animal Care and Use Committee, and in accordance with the AAALAC recommendations for the proper use and care of laboratory animals (protocol MO18M129, renewed as MO21M127). Four to six week old female Nude (002019), or CD1 (022) or C57BL6 (027) mice were purchased from Charles River, USA. Isoflurane anesthesia was used during imaging. The health conditions were monitored daily and/or criteria under which early euthanasia of an animal from the study was implemented included: general signs of distress such as hunched posture, lethargy, anorexia, dehydration, rough hair coat and/or those that are directly related to the experimental procedures e.g. loss of weight >10%, lethargy, restricted movement of limbs, distended abdomen. All tumor-bearing animals were sacrificed upon reaching pre-specified endpoints excepting those that reached the end of the study (see Kaplan-Meier), whereupon they were euthanized. Animals in distress were euthanized by carbon dioxide asphyxiation at the earliest possible time on the same day, and cervical dislocation was used to ensure death. This is an acceptable form of euthanasia for mice and in compliance with the recommendations of the Panel on Euthanasia of the American Veterinary Medical Association.

### Electroporation (EP) of 4UbFL expression plasmid

A patch of CD1 mouse leg was shaved of hair and 10 μg 4Ub-FL plasmid in 20 μL of PBS was injected into the *quadriceps femoralis* muscle followed immediately by injection of the 2 Needle Array to 5 mm depth encompassing the injection site and square wave EP (ElectroSquarePorator 833, BTX-2 Needle array 5mm gap, Harvard apparatus) delivered as eight pulses at 106 V for 20 ms with 200 ms intervals. One day post-EP, mice were anesthetized with isoflurane, injected IP with luciferin (0.3 mg in 100 μL water) and optical imaging was performed to determine basal level luciferase expression. Images were acquired for 10 min with a Xenogen IVIS 200 (Caliper, Hopkinton, MA). Equally sized areas were analyzed using Living Image 2.20 software.

### Syngeneic model

Female C57BL6 mice were inoculated IP with 3x10^5^ ID8-Defb29/VegfA-luc cells [38] in 100 μL PBS. At day 3, mice were imaged for basal level luminescence activity with a Xenogen IVIS 200 after IP injection with luciferin (0.3 mg in 100 μL water). Mice were randomized into groups and treated IP with compounds or vehicle (25% (*w*/*v*) β-Hydroxypropylcyclodextrin in water), and imaged again on days indicated.

### Spontaneous GEMM

To induce IP tumor formation in immunocompetent mice, oncogenes (shp53, AKT, c-Myc), luciferase and the Sleeping Beauty (SB) transposase (10 μg/plasmid) were diluted in 500 μL of PBS and injected IP into female C57BL/6 mice [39]. The mice were anesthetized by intramuscular injection of 50–100μL of a cocktail of ketamine 16 mg/mL / 3 mg/mL xylazine. The plasmid-injected mice were electroporated (EP) by the BTX ECM 830 square wave EP generator (BTX) (5 pulses, 200 V for 100 ms/pulse, 100 ms intervals between each pulse) with the caliper electrode (BTX) held across the waist of mouse. The mice were followed by IVIS imaging weekly for tracking tumor growth. The mice were sacrificed when the bioluminescence signal reached 10^9^ photons/sec/cm^2^/sr, or had enlarged abdomens due to the production of ascites, or had health conditions and/or criteria under which early euthanasia was required by the protocol. To track peritoneal tumor growth, bioluminescence imaging was performed by the IVIS Spectrum2000 (PerkinElmer) as the plasmid expressing SB transposase also produces luciferase. Briefly, the mice were injected IP with D-luciferin (GoldBio). After 10 min, the mice were anesthetized with isoflurane, and imaged by the IVIS Spectrum under the auto exposure mode. Bioluminescence in the peritoneal region was quantified as total photon counts/sec/cm^2^/sr using Living Image 3.0 Software (Perkin Elmer).

### Statistical analyses

Results are summarized via descriptive statistics (mean ± standard deviation (s.d.), median, range, frequency or percentage). Group differences in continuous outcomes were assessed via Wilcoxon rank sum test for pairwise group comparisons, or via Kruskal-Wallis test for global difference among three or more groups. For assessing the group differences in tumor growth and weight change, the fold change relative to baseline at the latest recorded time within 3 weeks of the treatment were compared among/or between groups, due to inferior survival rate in control arm which resulted low record-frequencies. Specifically, spaghetti plots of tumor and weight outcomes were provided across available time points with smoothing curves estimated from fitting a linear or quadratic polynomial function of time to illustrate the trend differences. Survival was summarized using Kaplan–Meier methods and group differences were compared using log-rank tests. In the case of an outcome with multiple group comparisons, the p-values were further adjusted via Holm approach [40]. The analyses were conducted using Prism (V.7 Graphpad, San Diego, CA) or R version 4.2.2 (R Foundation for Statistical Computing, Vienna, Austria) with the level of significance set at p≤0.05. Combination indices (CI) were calculated using Synergy finder (https://synergyfinder.fimm.fi/).

### Pharmacokinetics

For single dose pharmacokinetic studies, plasma samples (40 μL) were mixed with 200 μL of a water-methanol (1:9, v/v) containing the internal standard elacridar at 80 ng/mL. After mixing by pipetting and centrifuging for 4 min at 5500 x *g*, 0.25 μL of the supernatant was injected into LC-MS/MS system. Chromatographic separation was achieved with a Discovery HS F5 50 x 2.1 mm, 3μm; Supelco Inc. column at 30°C using gradient elution over a 2.2 min analytical run time. Mobile phase A was acetonitrile/water (5:95, v/v) containing 1% formic acid, and mobile phase B was acetonitrile containing 0.1% formic acid. The gradient was initiated with mobile phase B at 10% with a flow rate of 0.4 mL/min, increased to 100% over 0.9 min and then and held for 0.1 min, and returned back to 10% mobile phase B and allowed to equilibrate until 2.2 min. An SCIEX 5000 triple quadrupole mass spectrometer operated in positive electrospray ionization mode was used for the detection of RA475. The lower limit of quantitation (LLOQ) in plasma was 10 ng/mL.

For the stability studies and biodistribution studies, plasma or tissue homogenate (20 μL) was extracted with 100 μL of acetonitrile containing the internal standard RA413S (a structural analogue) at 10 ng/mL. Tissue homogenates were prepared at a concentration of 200 mg/mL in PBS. After vortex-mixing and centrifuging for 5 min at 2500 xg, 10 μL of the supernatant was injected into LC-MS/MS system. Chromatographic separation was achieved with a Zorbax XDB C18 (50 x 2.1 mm, 3.5μm; Agilent) column at room temperature using gradient elution over a 4.1 min analytical run time. Mobile phase A was water containing 0.1% formic acid, and mobile phase B was acetonitrile containing 0.1% formic acid. The gradient started with mobile phase B at 20% and was held at 20% for 0.5 minutes with a flow rate of 0.3 mL/min, then increased to 100% over 0.5 minutes and held for 2.0 minutes, and finally returned back to 20% mobile phase B and allowed to equilibrate until 4.1minutes. An AB Sciex 5500 triple quadrupole mass spectrometer operated in positive electrospray ionization mode was used for the detection of RA475. The lower limit of quantitation (LLOQ) in plasma was 10 ng/mL.

### Pharmacokinetic method analysis

Pharmacokinetic parameters were calculated from mean concentration-time data using non-compartmental methods in Phoenix WinNonlin version 8.3 (Certara, Princeton, NJ). Concentrations after the time 0 sample that were below the LLOQ were imputed with ½ that value (1 ng/mL). Any concentration that was determined to be an outlier by the Grubbs’s outlier test were excluded from analysis. The maximum plasma concentration (C_max_) and time to C_max_ (T_max_) were the observed values. The AUC_last_ was calculated using the log-linear trapezoidal method. AUC was extrapolated to infinity (AUC_INF_) by dividing the last quantifiable concentration by the terminal disposition rate constant (λ_z_). The λ_z_ was determined from at least 3 points on the slope of the terminal phase of the concentration-time profile. The terminal half-life (T_1/2_) was determined by dividing 0.693 by λ_z_. Clearance (Cl) or apparent Cl (Cl/F) was calculated by dividing the dose administered by AUC_INF_. Volume of distribution (V) or apparent V (V/F) was calculated by dividing Cl or Cl/F by λ_z_. If the percent AUC extrapolated was >20% or the r^2^ of λ_z_ was <0.9, the AUC_INF_, Cl/F, T_1/2_ and V/F were not reported. The oral and IP bioavailability was calculated as:

$$\text{F}\;(\%)=\left\{\text{Dose}\;\text{IV}\times \;\text{AUCINF}\;\text{PO}/\text{DosePO}\times \text{AUCINF}\;\text{IV}\right\}\;.100\%$$

The Method of Bailer was used to estimate the variance of AUC given the calculated variance of the mean concentration at each time point [41]. A pairwise comparison utilizing Z test was used to determine whether there was a significant difference between RA475 exposures as expressed by AUC _last_ [42]. Additionally, the Up284 exposure was truncated to 8 h and compared to the same route of administration and exposure as RA475. In all cases, p < 0.05 was considered statistically significant.

## Results

### Evolution of RA475

Modifications of Up284 were sought to improve its drug-like properties without significant compromise of its activity in vivo, and several new compounds were synthesized (S1 File). Based on our prior studies of its pharmacophore, the central ring nitrogen was the most logical for addition of charged moieties, including RA475 and RA484. We also examined a change to either 3,4 spiro rings (RA477, RA479), or addition of 3-Fluoro substitutions (RA482) (S1 File). Our focus is developing iRPN13 to treat ovarian cancer resistant to standard of care drug regimens. Thus, as an initial screen of their cytotoxicity, a pair cell lines derived from an ovarian cancer patient either pre- or post-treatment (PEA1 and PEA2) was treated with each compound or Up284 for 72 h and IC_50_ determined by MTT assay. These compounds exhibited similar cytotoxicity to each other, and in PEA1 and PEA2, although they were half as potent as Up284 in this in vitro assay (S1A Table).

We elected to focus on RA475 and expanded the analysis in a larger panel of cell lines including those from common cancer types and normal tissues. RA475 demonstrated potency against the growth of most cancer types, suggesting broad applicability, but relatively spared normal cells, implying a significant therapeutic window (S1B Table). This panel included another pair of cell lines isolated from another ovarian cancer patient with drug-resistant disease (PEO4) and pre-treatment (PEO1) [10], and both were similarly sensitive to RA475. Overall, RA475 was approximately 3-fold less potent than Up284 (mean IC_50_ across all cancer lines in S1B Table of 0.42 μM for RA475 vs. 0.12 μM for Up284).

A wash-out experiment was performed to assess reversibility of the toxicity. PEA1 cells were treated with RA475 for 1 h before the medium was replaced with fresh culture medium, and the cells were then allowed to grow for another 71 hours (PEA1-WO). In parallel, PEA1 cells were incubated with RA475 continuously for 72 h. When cell viability was measured using MTT assay, it was evident that washing out RA475 reduced its cytotoxic effect on PEA1 cells (S1 Fig). Possible reasons include slow uptake of RA475, reversible binding and neutralization by antioxidants in the medium.

### RA475 cytotoxicity is mildly synergistic with cisplatin

To study the effect of RA475 in combination with approved therapeutics (DNA damaging agents or radiation) an MTT assay was performed using various concentrations of both drugs. Briefly PEA1 and OVCAR3 cells were each treated with drug combination (RA475 plus cisplatin) for 72 h in a checker board analysis and cell viability was measured using MTT assay. The data were analyzed and plotted using the Synergy Finder web application. RA475 demonstrated synergistic cytotoxicity with cisplatin against both cell lines (S2 and S3 Figs).

### RA475 induces apoptotic cell death

**Fig 2:**
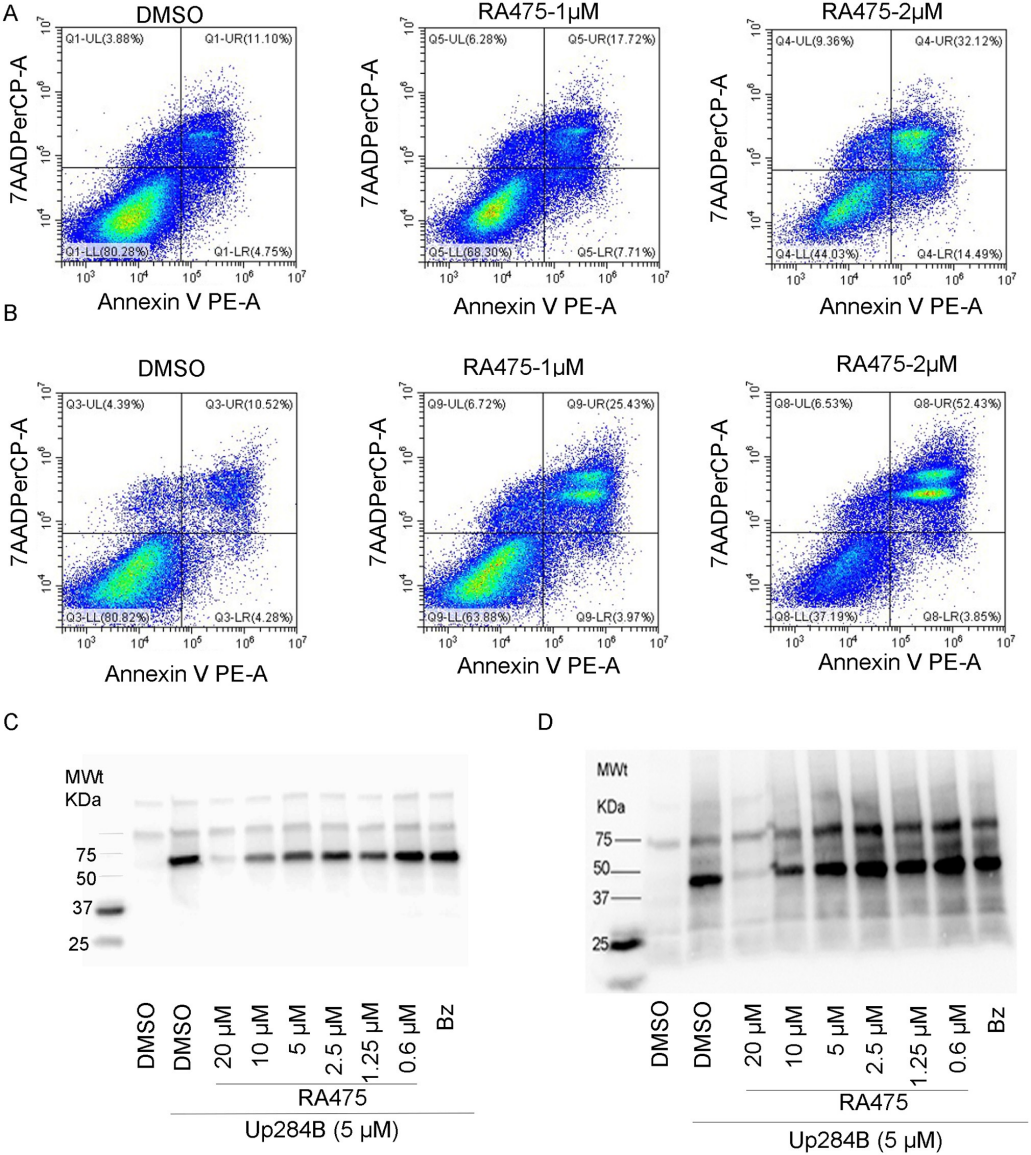
RA475 binds to RPN13 and induces rapid apoptosis. Two human ovarian cancer cell lines, (**A**) ES2 and (**B**) OVCAR3, were treated with DMSO vehicle alone or 1 μM or 2 μM RA475 and 16 h later the cells were harvested and stained with 7-AAD and Annexin V. Flow cytometric analysis showed a dose-dependent induction of 7-AAD and Annexin V double positive cells after treatment with RA475. **C, D**) RA475 competition labelling experiment. **C**) ES2 **D**) SKOV3 clarified cell lysate (40 μg/sample) in MPER buffer was pretreated with streptavidin-coated magnetic beads for 45 min at 4°C to deplete non-specific biotinylated proteins in the cell lysate. The beads were removed, and 40 μL of the pre-cleared cell lysate was incubated with RA475 at indicated concentrations or bortezomib (1 μM) for 45 min at 4°C, and then treated with Up284B (5 μM) for 45 min at 4°C as indicated. Next, the samples were mixed with Laemmli sample buffer (BioRad) and boiled for 5 min. The proteins were separated using a 4–15% Bio-Rad Mini-PROTEAN SDS-PAGE gel (1 h at 100 V), and transferred to PVDF membrane overnight at 4°C (24 V). The membrane was blocked with 5% BSA in PBST for 1 h at RT and washed for 20 minutes (3X with PBST). The membrane was then probed with HRP-streptavidin (1:10,000 in PBST) for 1 h at RT, washed for 30 min (3X with PBST), and developed using HyGLO chemiluminescent detection reagent (Denville) for biotin detection.

To determine the mode of cell death, ES2 and OVCAR3 ovarian cancer cell lines were treated with 0, 1 or 2 μM RA475 for 16 h, and stained with 7-AAD and Annexin V-FITC to assess cell viability and apoptosis (Fig 2A and 2B). Flow cytometry showed that RA475 increased the percentage of 7-AAD/Annexin V positive cells dose dependently compared to a DMSO (vehicle)-treated group in both lines. Progression to cell death over 24 h to 48 h was confirmed by analysis of PARP cleavage (S4 Fig) and morphologically (S5 Fig). Likewise, RA475 induced apoptosis in the triple negative breast cancer line MDA-MB-468 (S6 Fig). This is consistent with Up284 and other prior iRPN13 [37].

### RA475 competes covalent binding of Up284B to RPN13

Biotinylated Up284 (Up284B) covalently binds to RPN13 in direct labeling assays in vitro, and Up284 also competes with biotinylated prototype iRPN13, RA190B, for binding to RPN13 [37]. Since RA475 cannot be biotinylated on the central ring nitrogen like RA190 or Up284, we resorted to a competition assay. To elucidate whether RA475 binds to RPN13, we used Up284B as a probe and performed the labeling assay after treating ovarian cancer cell lysates (ES2 and SKOV3) in the presence of titrated doses of RA475 (unlabeled). We observed that RA475 efficiently competes with Up284B for binding to RPN13 (Fig 2C, ES2 and Fig 2D, SKOV3) dose dependently, whereas bortezomib had no impact (negative control).

### RA475 induces accumulation of very high molecular weight polyubiquitinated proteins

**Fig 3:**
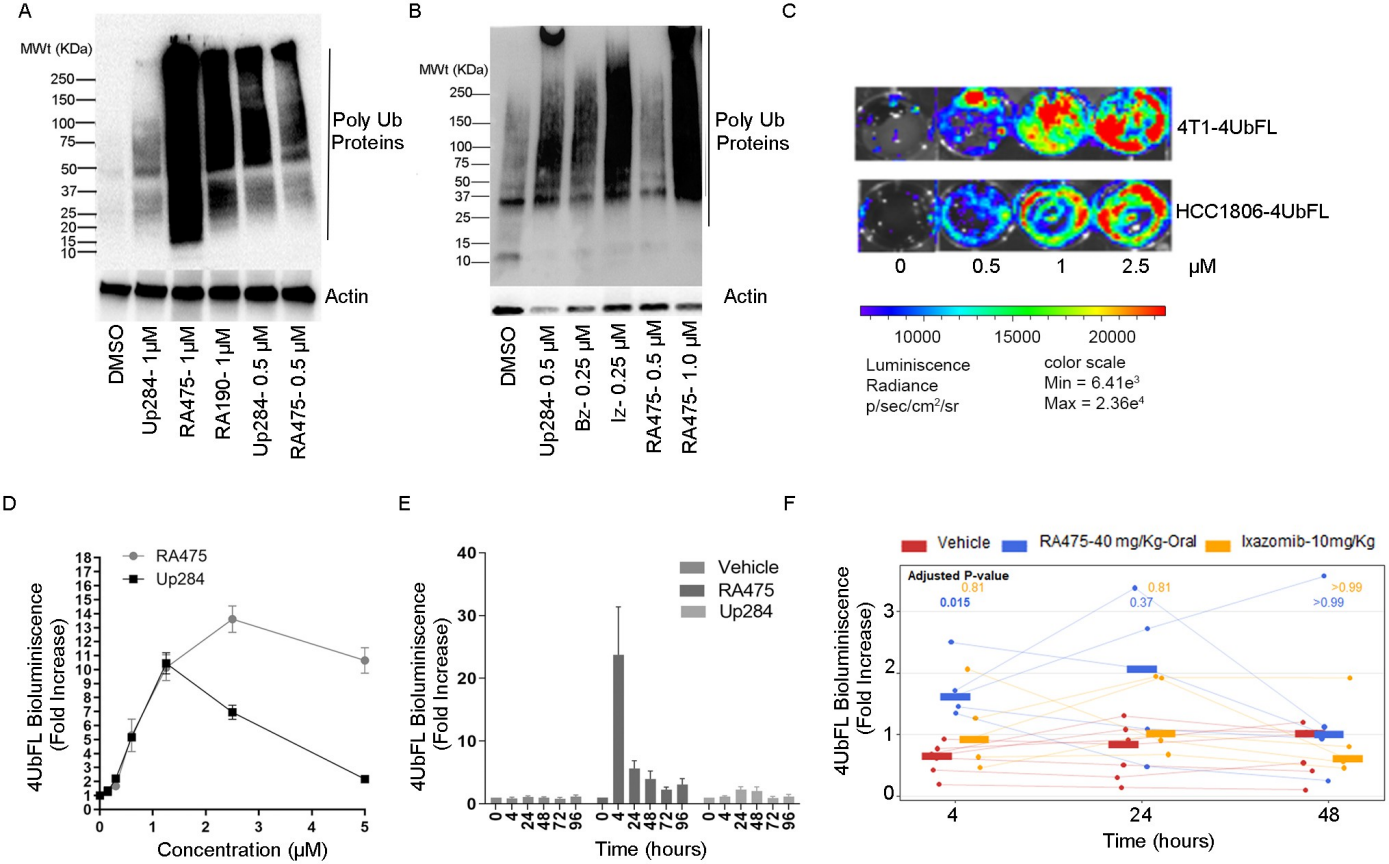
Impact of RA475 on proteasome function. **A)** RA475 triggers the accumulation of high molecular weight polyubiquitinated proteins in SKOV3 cells treated with Up284 (0.5 μM and 1 μM), 1 μM RA190, RA475 (0.5 μM and 1 μM) or vehicle alone (DMSO) using anti-Ubiquitin antibody by immunoblot analysis. Actin was used as loading control. **B)** RA475 triggers the accumulation of high molecular weight polyubiquitinated proteins in OVCAR3 cells treated with Up284 (0.5 μM), bortezomib (Bz 0.25 μM), ixazomib (Iz 0.25 μM), RA475 (0.5 μM or 1 μM), or vehicle alone (DMSO). **C)** 4T1 and HCC1806 cells stably expressing 4UbFL were treated with compounds at indicated doses for 4 h and imaged by IVIS200 after the addition of luciferin substrate (50 μL, 7mg/mL in PBS). **D)** Sub-confluent cultures of ES2 cells stably expressing 4UbFL were seeded at 10,000 cells/well in 96-well microtiter plates. At 18 h post transfection, cells were treated with compounds or vehicle (DMSO) at the doses indicated. After a 4 h incubation, the cells were lysed and the luciferase activity in each cell lysate was determined with a luciferase assay kit (Promega) according to the manufacturer’s instructions. Bioluminescence was measured by using a luminometer (Glomax Multidetection system, Promega) **E)** A patch was shaved of hair on one leg of CD-1 mice, and 10 μg 4UbFL plasmid in 20 μL of PBS was injected into the *quadriceps femoralis* muscle followed immediately by injection of the 2 Needle Array to 5 mm depth encompassing the injection site and square wave electroporation (ElectroSquarePorator 833, BTX-2 Needle array 5mm gap, Harvard apparatus) delivered as eight pulses at 106V for 20 ms with 200 ms intervals. One day post electroporation, mice were anesthetized with isoflurane, injected IP with luciferin (0.3 mg in 100 μL water) and optical imaging was performed to determine basal level luciferase expression. Images were acquired for 10 min with a Xenogen IVIS 200 (Caliper, Hopkinton, MA). Equally sized areas were analyzed using Living Image 2.20 software. Mice were imaged weekly during treatment. Mice were randomized into three groups (n = 5) and treated IP once with Vehicle (25% (*w*/*v*) β-Hydroxypropylcyclodextrin in water, Sigma Aldrich) alone, RA475 (40 mg/Kg) or Up284 (40 mg/Kg) and mice were imaged again at 4 h, 24 h, 48 h, 72 h and 96 h later. **F)** Same as in **E** but RA475 (40mg/Kg) and ixazomib (10mg/Kg, recommended dose) were delivered orally. Thick line indicates median measurements. P-values are results based on Wilcoxon rank sum test for pairwise comparisons to vehicle cohort at each time point with multiplicity adjustment.

Accumulation of high molecular weight polyubiquitinated proteins (polyUb) is a classical phenomenon of proteasome function inhibition due to the stabilization of its substrate. In the ovarian cancer cell line SKOV3, RA475 treatment caused robust accumulation of polyUb in dose dependent manner, as seen with RA190 and Up284 which suggests a similar mechanism of action (Fig 3A). RA475 demonstrated the phenomenon in the ovarian cancer cell line OVCAR3. Importantly, like Up284, RA475 induced forms of polyUb that did not run into the gel, whereas bortezomib and ixazomib only induced the smaller poly Ub forms, typically <250kDa (Fig 3B). This difference likely reflects inhibition of proteasomal DUBs by RA475 and Up284 as well as 20S CP degradation, whereas bortezomib and ixazomib only inhibit the latter.

### RA475 stabilizes proteasome reporter protein 4UbFL in vitro

Firefly Luciferase (FL) is a stable protein in cells, but when fused with four molecules of ubiquitin (4Ub) it is targeted for rapid degradation by proteasomes. Inhibition of proteasome function, for example by iRPN13, stabilizes the 4UbFL protein and the corresponding increase in its level can be followed using bioluminescence imaging or a luminometer. Triple negative breast cancer cell lines 4T1 and HCC1806 stably transfected with 4UbFL plasmid exhibited dose dependent increases in bioluminescence signal when imaged 4 h after treatment with RA475 (Fig 3C). ES2 cells stably expressing 4UbFL also show a dose-dependent increase in bioluminescence after 4 h treatment with Up284 or RA475 as measured by a luminometer. Data are presented as fold increase over bioluminescence of cells treated with DMSO alone (Fig 3D). Reductions in 4UbFL signal at high drug concentrations reflects rapid cell death.

### RA475 stabilizes 4UbFL reporter in vivo

Tagged female CD-1 mice were electroporated after injection of the 4UbFL expression plasmid into the muscle of one leg. Administration of luciferin substrate IP and bioluminescense imaging of the mice at the site of 4UbFL plasmid injection was performed 24 h after electroporation. This time point was considered baseline. Mice were then randomized into three groups (n = 5) and treated with vehicle alone (25% β-hydroxypropylcyclodextrin-water solution), RA475 (40 mg/Kg) or Up284 (40 mg/Kg), each administered as a single IP dose. The mice were imaged again at 4h, 24h, 48 h, 72 h and 96 h later. Data are presented as fold increase over bioluminescence of injection site at baseline for individual mice followed longitudinally. RA475 treatment rapidly increased the bioluminescence from the leg muscle as compared to vehicle and the response was sustained over 96 h (Fig 3E). This is consistent with *in vitro* stabilization of 4UbFL with RA475 at 4 h (Fig 3D). Notably, in contrast to the in vitro findings (Fig 3D), a much weaker effect was observed with Up284, suggesting that it is not as bioavailable as RA475 in this context (Fig 3E). When RA475 was delivered as a single oral (PO) 40 mg/Kg dose, the stabilization of 4UbFL in muscle was much lower than seen for the single IV dose, although it was more effective than a single oral dose of a licensed 20S proteasome inhibitor, ixazomib, at its MTD of 10 mg/Kg (Fig 3F).

### RA475 stability in murine and human plasma and liver microsomes

As a prelude to pharmacologic studies of RA475, we first examined its stability in plasma and liver microsomes of both murine and human origin (S2 Table). RA475 did not degrade upon addition to murine or human plasma for 1 h at 37°C, since all alterations in the area ratios were well within typical analytical variability allowance (15%). There was no significant (<10%) degradation in mouse or human liver microsomes either with or without NAPDH over 1 h (S2 Table). By contrast, the first generation compounds RA190 and RA183 were more rapidly broken down in human and mouse microsomes [12].

### RA475 binding to mouse and human plasma proteins and albumin

To determine binding capabilities of RA475 to mouse plasma proteins, LC-MS/MS analysis was performed by spiking test compounds at concentration of 1 μM into plasma and dialyzing 5 h against buffer to seek equilibrium. RA475 showed >99.0% mouse plasma protein binding (S3 Table), 99.1% protein binding in human plasma (S4 Table) and 93.8% binding to human serum albumin protein (S5 Table).

### Experimental LogD and aqueous solubility of RA475

Successful in vivo studies require a fully soluble product. To assess their hydrophobic/hydrophilic properties, we first determined distribution coefficients (LogD) for Up284 and RA475 (and reference compound Mebendazole) in n-octanol versus phosphate-buffered saline (PBS), pH 7.4 using a shake-flask method and LC-MS/MS to assess compound distribution (S6 Table). The LogD for RA475 was 1.58, whereas for Up284 it was higher at 2.12.

Aqueous solubility of Up284 and RA475 in phosphate-buffered saline, pH 7.4 was determined as 37 μM and 137 μM, respectively (S7 Table). The calibration curves are shown in (S7 Fig) for the assays of kinetic solubility in phosphate-buffered saline, pH 7.4.

### RA475 reactivity with glutathione

We determined the glutathione (GSH) reactivity of RA475 and Up284 (and reference compound PCM-0102244) at five time points over 90 minutes using HPLC-MS (S8 Table). RA475 and Up284 readily and indistinguishably reacted with GSH within 30 min.

### Inhibition of human ether-a-go-go related gene (hERG) channel activity

In vitro hERG potassium channel function was inhibited by RA475 with an IC_50_ of 85.8 μM (S8 Fig) and by Up284 with an IC_50_ of 4.3 μM. The observed IC_50_ value of the positive control ligand (Haloperidol) was consistent with published data [13].

### RA475’s NOAEL in mice >80mg/kg for a single dose

RA475 in 25% β-hydroxy propyl cyclodextrin in water was administered at increasing single doses to CD-1 mice (n = 3/group) via intraperitoneal injection. No evidence of adverse events, including weight loss, was detected at or below 80 mg/Kg of animal weight, the highest dose level tested.

### Pharmacokinetics and tissue distribution of RA475 in mice

**Fig 4:**
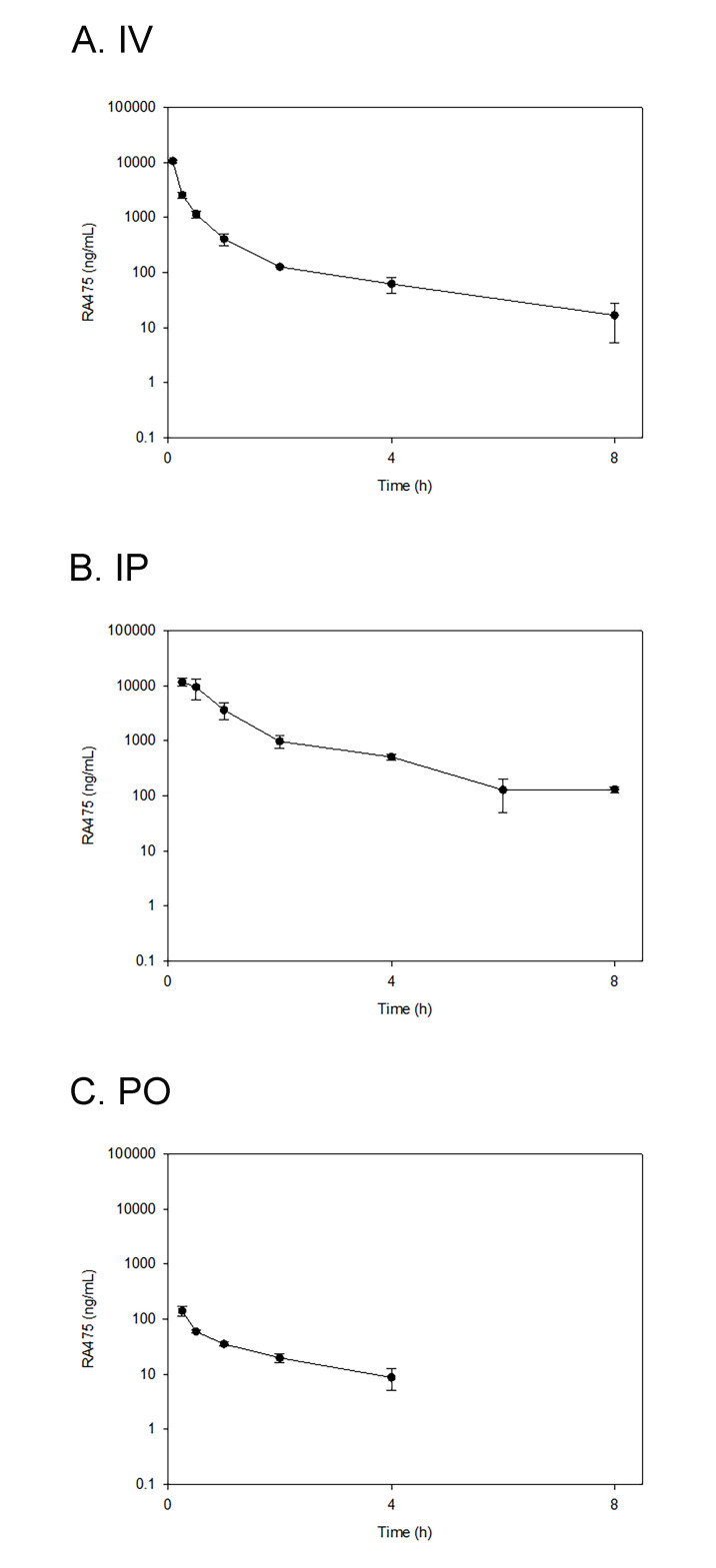
Pharmacokinetic studies. Male CD1 mice (n = 4/time point) were treated with RA475 IV (10mg/kg), IP (40mg/kg) or PO (40mg/kg). Blood was collected at indicated time points thereafter and analyzed for RA475 concentrations by LC-MS/MS.

CD1 (n = 4 per time point and condition; 8 weeks old, 26.8±3g) mice were administered a single dose of RA475, either IV (10 mg/kg), PO (40 mg/kg), or IP (40 mg/kg), and thereafter serial blood plasma samples were analyzed for RA475 levels by LC–MS/MS. The animals were randomly assigned to the treatment groups (S9 Table) and fasted for 4 h before dosing. Eight time points for IV and IP (0.083, 0.25, 0.5, 1, 2, 4, 6, and 8 h) and seven time points for PO (0.25, 0.5, 1, 2, 4, 6, and 8) administration were set for this pharmacokinetic study. Mice were injected IP with 2,2,2-tribromoethanol at the dose of 150 mg/kg prior to drawing the blood in microtainers containing K_3_EDTA via retro-orbital bleeding. Blood samples were centrifuged 10 min at 3000 rpm. All samples were immediately processed, flash-frozen and stored at -70°C until subsequent analysis. No obvious adverse effects were observed during this PK study. The average RA475 plasma concentrations data for IV, IP, and PO dosed groups (S10–S12 Tables) is graphically presented in Fig 4, and may be compared to published data for Up284 [37]. Pharmacokinetic parameters were calculated from mean concentration-time data using non-compartmental methods in Phoenix WinNonlin version 8.3 (Certara, Princeton, NJ) **(**S13 Table). Using the Method of Bailer, there was no difference in dose normalized AUC_last_ by any route of administration of RA475. The dose normalized AUC_8h_ for RA475 was significantly lower than the AUC_8h_ for Up284 via the IP route of administration. In summary, the calculated oral and IP bioavailability for RA475 was 1.1% and 103% respectively (S13 Table), which contrasts values of 23% and 87% respectively for Up284 [37], suggesting the guanine moiety produces a profound reduction in the oral bioavailability of RA475. This is consistent with the minimal stabilization of UbFL after oral delivery of 40 mg/Kg RA475 (Fig 3F), and compared to the robust (>20-fold) stabilization observed upon 40 mg/Kg delivered IP (Fig 3E), or *in vitro* with 1.25 μM of either RA475 or Up284 (Fig 3D).

**Fig 5:**
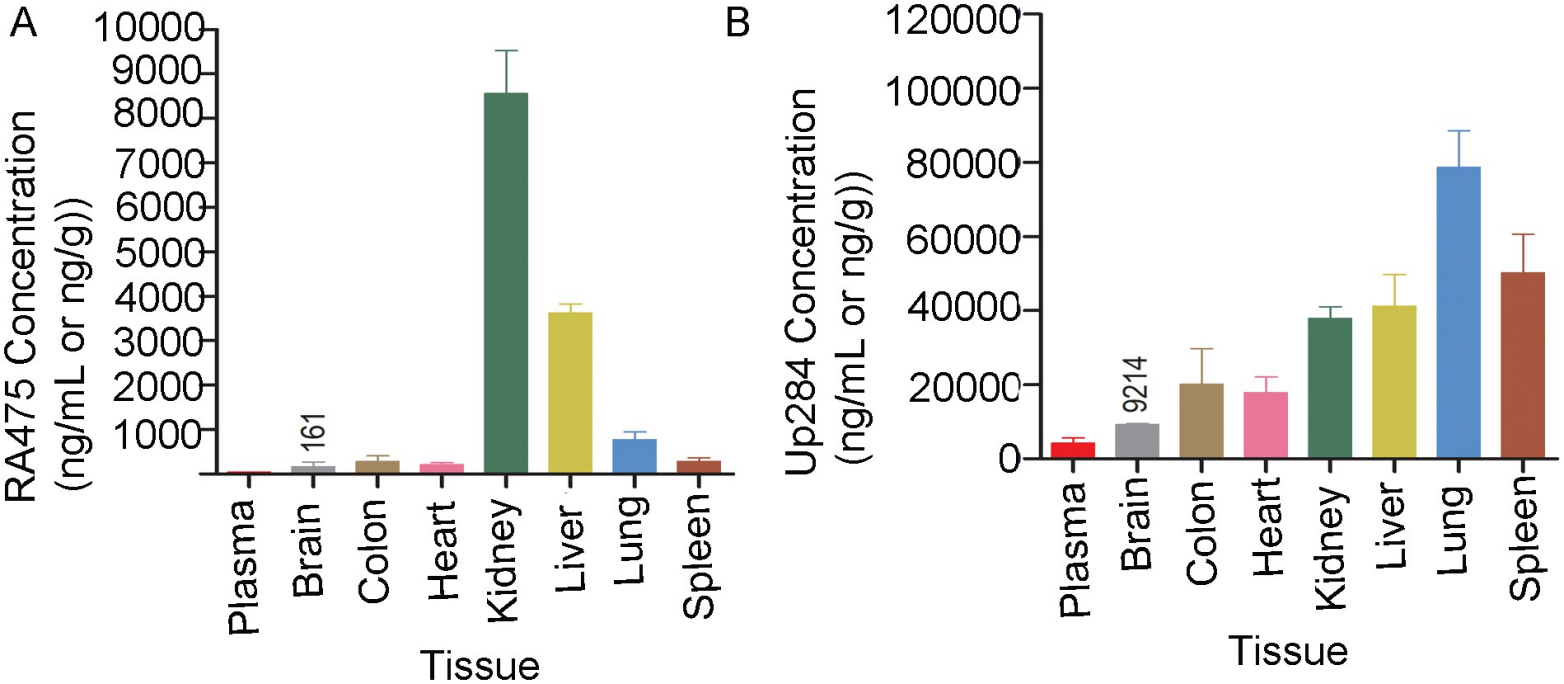
Tissue distribution of RA475 and Up284 in mice. Female CD1 mice (n = 3/drug) were treated with one IV dose of **A)** RA475 (40mg/kg) or **B)** Up284 (40mg/Kg) and euthanized after 24 h with collection of plasma and tissues including brain, colon, heart, kidney, liver, lung and spleen. RA475 and Up284 concentrations in tissue samples were analyzed by LC-MS/MS.

In a separate experiment to assess drug distribution, CD1 mice were injected with one dose of RA475 (40 mg/kg, IV) and tissues collected after 4 h and 24 h. RA475 levels were analyzed in all major tissues (brain, colon, heart, kidney, liver, lung and spleen) by LC-MS/MS. RA475 accumulated primarily in kidneys and liver, and to a lesser extent lung at 24 h (Fig 5).

### RA475 treatment of ovarian tumor in a syngeneic mouse model

**Fig 6:**
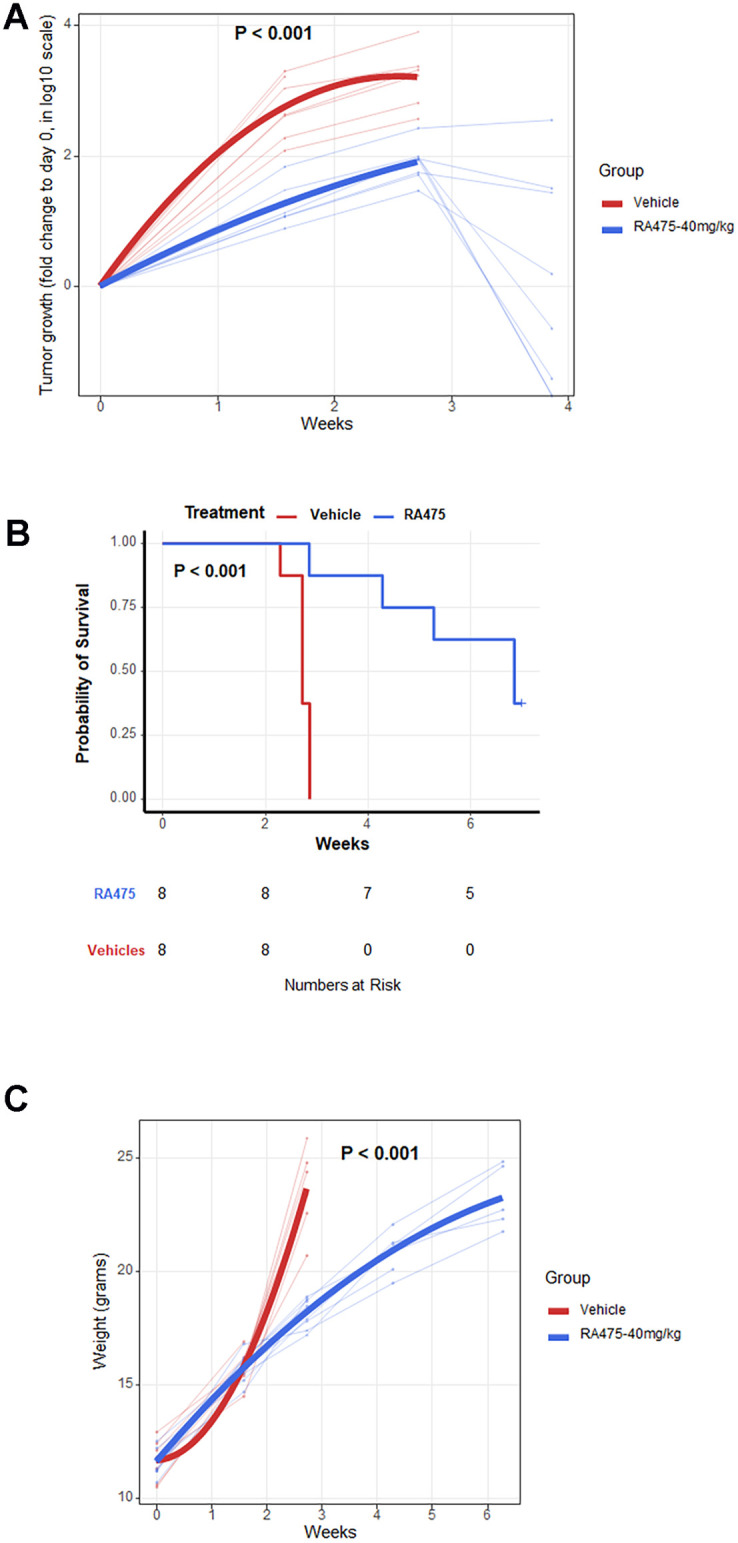
Treatment with RA475 of mice bearing an orthotopic syngeneic ovarian cancer model tumor. Female C57BL6 mice bearing ID8-Luc tumor were monitored for tumor burden by IVIS imaging **(A)**, survival **(B)** and animal weight **(C)** after treatment with IP treatment every three days with RA475 (40mg/Kg, n = 8) or vehicle alone (n = 7). To initiate the study, the mice were injected IP with ID8-Vegf Defb29 cells stably expressing firefly luciferase (0.5 X 10^6^ cells in 100 μL PBS). After 72 h, mice were imaged after administration of luciferin (100 μL, 7.8 mg/mL) for their basal luminescence levels using an IVIS200 imager. Mice with visible luminescence were randomized into two groups and treated with Vehicle alone (25% β-hydroxy propylcyclodextrin in water, 200 μL, every 3 days, IP) or RA475 (40 mg/Kg, every 3 days, IP). Mice were imaged every week to measure bioluminescence (photon counts/sec/cm^2^/sr) expressed by tumor burden. In the vehicle group all 7 mice showed enlarged abdomens due to ascites formation.

In addition to direct tumor cell killing, it is possible that RA475 may trigger an immunogenic cell death and thus anti-tumor immunity that could contribute to tumor control. Therefore, we elected to first test the treatment potential of RA475 in an ovarian tumor in a syngeneic mouse model. Since ovarian cancer is shed into, and spread within the peritoneal cavity, female C57BL6 mice were injected IP with ID8-Vegf Defb29 cells stably expressing firefly luciferase (0.5 x 10^6^ cells ID8-luc) in PBS (100 μL). After 72 h, mice were imaged for their basal luminescence levels using an IVIS200 imager to confirm the presence of viable tumor cells. Mice with visible luminescence were randomized into two groups and treated with Vehicle (25% β-hydroxy propylcyclodextrin in water, 200 μL, every 3 days, IP) and RA475 (40 mg/Kg, every 3 days, IP). Mice were weighed and, after administration of luciferin (100 μL, 7.8 mg/mL), imaged every week to assess bioluminescence associated with tumor burden. In the vehicle group all 7 mice rapidly developed enlarged abdomens due to ascites formation. The mice (n = 8) treated with RA475 (40mg/Kg every 3 days, IP) show tumor control (Fig 6A), prolonged survival (Fig 6B), and continue to gain weight (Fig 6C) while on treatment.

### Spontaneous Genetically-Engineered Mouse Model (GEMM) of primary peritoneal/ovarian cancer

**Fig 7:**
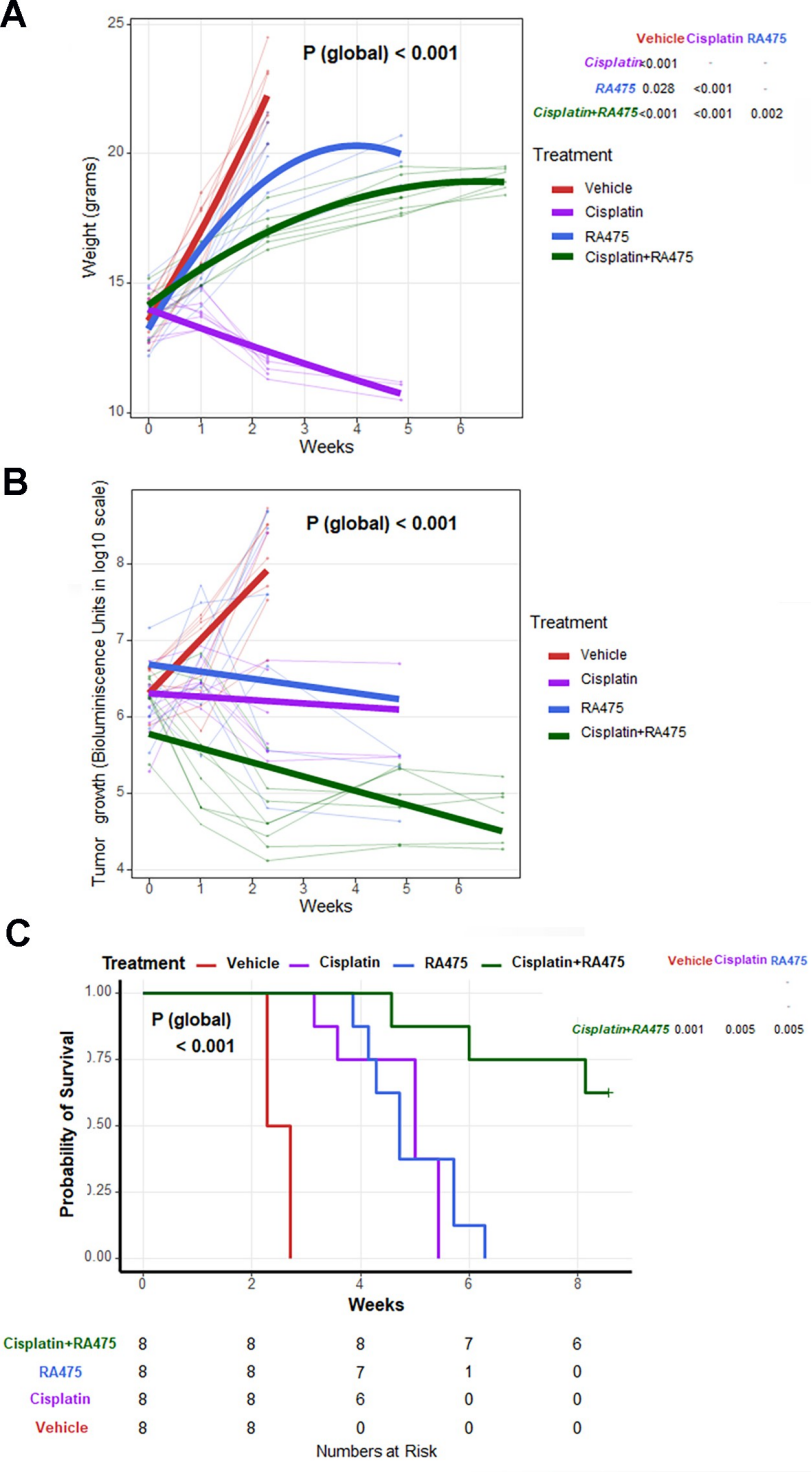
Administration of RA475, cisplatin or the combination to mice bearing spontaneous, genetically-engineered model ovarian/peritoneal cancer. Female C57BL/6 mice were co-injected IP with sleeping beauty vector and plasmids expressing carrying shp53, AKT, c-Myc and firefly luciferase genes, followed by electroporation (EP) to deliver these plasmid DNA into the peritoneal cavity cells wherein they integrate randomly into their genome [39]. After 7 days mice were imaged by Xenogen IVIS200 for bioluminescence and randomized. Four groups (n = 8) were treated IP with vehicle alone or RA475 (40mg/kg, IP, every 3 days for a total of 12 doses) or cisplatin (5 mg/Kg, IP, once a week for a total of 5 doses), or combination of RA475 (40mg/Kg, IP, every 3 days for a total of 12 doses) plus cisplatin (5 mg/Kg, once a week for a total of only 3 doses), and followed for animal weight (**A**), tumor burden by luminescent imaging as photon counts/sec/cm^2^/sr (**B**), and survival (**C**).

GEMM better mimic the spontaneous development of cancers in an immune competent host than transplantable tumor lines. Female C57BL/6 mice were co-injected IP with sleeping beauty vector and plasmids expressing carrying shp53, AKT, c-Myc and firefly luciferase genes, followed by electroporation (EP) to deliver these plasmid DNA into the peritoneal cavity cells wherein they integrate randomly into their genome [14]. After 7 days mice were imaged by IVIS200 for bioluminescence and randomized. Four groups (n = 8) were treated IP with vehicle or RA475 (40mg/kg, IP, every 3 days) or Cisplatin (5 mg/Kg, once a week) or the combination of RA475 (40mg/Kg, IP, every 3 days) plus Cisplatin (5 mg/Kg, once a week). RA475 treatment alone (12 doses) delayed but did not significantly reduce tumor burden. As compared to RA475 alone, treatment with cisplatin alone (5 doses) significantly better delayed, but did not reduce, tumor burden (Fig 7A and 7B). The RA475 treatments extended survival to a similar extent as cisplatin alone (Fig 7C). While cisplatin reduced tumor growth, it led to severe toxicity including weight loss >20% that was not evident with RA475 alone. For the combination, cisplatin was delivered total of 3 doses (once a week) and RA475 given as 12 doses (every 3 days) which resulted in tumor regression based on bioluminescence (Fig 7B), and significantly extended survival (Fig 7C), consistent with in vitro evidence of their synergy (S2 and S3 Figs).

## Discussion

The prototypic iRPN13 candidate, RA190, is active against several solid cancer models and multiple myeloma but it has several features unsuitable as a drug [3–7]. By substitution of its PAINS-like structure with a spiro backbone we developed new iRPN13s including Up284 and RA475 with potentially improved specificity. RA475 retains the spiro backbone of the precursor Up284 but its guanidine moiety was intended to enhance solubility, and other key drug properties. RA475 has lower cLogP (1.751 vs. 2.651) and log D (1.58 vs. 2.12) values compared to Up284, and improved aqueous solubility (137 μM vs. 37 μM). RA475 shows weaker in vitro inhibition of hERG than Up284 (IC50 of 85.8 μM vs. 4.3 μM), potentially reducing risk of cardiotoxicity.

In healthy animals Up284 has limited access to the brain, while RA475 has relatively even lower accumulation in brain tissue (Fig 5) which suggests lower potential for neurotoxicity. Up284 may be more suited to treat brain cancers, although they are often associated with degradation of the blood-brain barrier. Bortezomib concentrations in human cerebrospinal fluid and murine brain tissue reach only 5–7% of serum concentrations, and at therapeutic doses this does not impair cognition [43]. Carfilzomib and ixazomib are also mostly excluded from the brain [44], although cases of posterior reversible encephalopathy syndrome (PRES) have occurred in patients receiving ixazomib and bortezomib [45].

Since RA475 competes with the adduct formation of Rpn13 by Up284B, RA475 presumably binds to Cys88. Cys88 resides in a groove that is critical for the attachment of RPN13 to the 19S proteasome subunit where it serves as a substrate receptor for the proteasome. However, more work to understand its mechanism of action is needed, and confirm that RPN13 is its critical target in vivo.

The gene encoding RPN13, *ADRM1*, has been proposed as an oncogene in ovarian cancer [8, 9]. Since RA475 targets RPN13, it has potential as a precision medicine for treatment of ovarian cancer patients. However, the in vitro cytotoxicity assays suggest that these iRPN13 have activity against diverse solid and liquid cancer types.

RPN13 inhibition results in rapid buildup of polyUb proteins, ER stress and consequent apoptotic cell death in cancer cells (Figs 2, 3A and 3B). Prior studies with iRPN13 analogs show they trigger surface display of calreticulin, a hallmark of immunogenic cell death, enhance antigen presentation via MHCI and induce antitumor immunity. This suggests their potential in combination with immune checkpoint inhibitors. The iRPN13 may differ in this regard to the licensed 20S CP inhibitors, in particular because they induce greater amounts of protein aggregates that include much higher molecular weight polyUb. We speculate that these polyUb aggregates likely include tumor antigens and can be readily processed by phagocytic antigen presenting cells. Another difference is that RPN13 is not a component of immunoproteasomes that function in antigen presentation, whereas the licensed proteasome inhibitors do inhibit their function.

While the on-target activity (i.e. stabilization of 4UbFL) of RA475 is very similar to its precursor Up284 *in vitro* (Fig 3D), in vivo RA475 appears superior to Up284 (Fig 3E) with a rapid effect when delivered IP. However, in contrast to Up284, RA475 did not appear effective when delivered orally, and this is supported by the pharmacologic data showing extremely poor oral bioavailability.

Preclinical efficacy studies in ovarian cancer models with RA475 closely mirror those with its precursor Up284 when delivered IP, the most potent route for treatment of ovarian cancer patients. RA475 demonstrated superior activity to Up284 (Fig 2E) in a leg muscle-localized proteasome dependent reporter assay when it was delivered IP to mice, possibly due to RA475’s stability in liver microsomes (93% present after 1 h, compared to only 52% for Up284). The dose-normalized AUC_8h_ is not significantly different between RA475 and Up284 but the dose-normalized C_max_ is 2.3-fold higher for RA475 suggesting C_max_ rather than total exposure may be driving the pharmacological effect. However, RA475 is more rapidly cleared from the plasma than Up284 when administered IV.

The addition of the guanidine moiety enhanced solubility allowing formulation in 25% hydroxypropylcyclodextin-water (β-HPCD), which is a widely used excipient. RA475 powder dissolves directly into 25% β-HPCD for IV or IP delivery. Chemical synthesis of RA475 is simple (three step) from commercially available starting materials and readily synthesized in bulk.

In sum, after multiple rounds of development, RA475 and Up284 have emerged as more drug-like iRPN13 candidates with promising therapeutic activity, but distinct pharmacologic properties. Of particular interest in ovarian cancer is their similar activity against cell lines from pre-treatment and treatment resistant disease from the same patient. Platinum-based chemotherapy is the first line treatment for ovarian cancer, and it is encouraging that RA475 showed synergy with cisplatin, in vitro and in vivo.

The failure of 20S proteasome inhibitors against solid tumors may reflect poor drug access (Fig 1D–1F). Our data suggest that targeting RPN13, a ubiquitin receptor within the 19S proteasome subunit, with our inhibitor RA475, that is not based on a peptide backbone, may overcome this limitation.

## Supporting information

S1 Fig Reversibility of RA475 by wash out. PEA1 cells were treated with RA475 for 72 h (PEA1) and in another set PEA1 cells were treated with RA475 for 1 h, and then RA475 was washed out (WO) by replacing the medium with fresh medium and cultured for an additional 71 h (PEA1-WO). Cell viability was measured using MTT assay and the data was plotted percent of control.
(TIF)

S2 Fig Analysis for synergy of cytotoxicity of cisplatin and RA475 in PEA1 cells. Human ovarian cancer-derived PEA1 cells in a 96 well format were treated with combinations of RA475 and cisplatin in a checker board assay format. The cells were then incubated for 72 h and cell viability was measured using MTT assay. Data were analyzed and plotted using the Synergy Finder web application. RA475 demonstrated mild synergy with cisplatin for cytotoxicity for PEA1 cells.
(TIF)

S3 Fig Analysis for synergy of cytotoxicity of cisplatin and RA475 in OVCAR3 cells. Human ovarian cancer-derived OVCAR3 cells in a 96 well format were treated with combinations of RA475 and cisplatin in a checker board assay format. The cells were then incubated for 72 h and cell viability was measured using MTT assay. Data were analyzed and plotted using the Synergy Finder web application. RA475 demonstrated mild synergy with cisplatin for cytotoxicity for OVCAR3 cells.
(TIF)

S4 Fig Induction of PARP cleavage in OVCAR3 and ES2 cells by RA475. Human ovarian cancer-derived OVCAR3 or ES2 cells were seeded in a 10 cm dish at a density of 750,000 cells in 10 mL of RPMI growth medium. The following day, the cells are treated with compounds at the specified doses for 48 hours. The medium was then aspirated, and the cells are trypsinized, pelleted, washed with PBS, and pelleted again using a centrifuge. The cell pellet is lysed with 200 μL of RIPA buffer containing 2 μL of protease inhibitors at 4°C and sonicated (Misonix, 50 Amplitude) for 20 seconds in three cycles at 4°C. The lysates were centrifuged at high speed for 2 minutes to remove cell debris, and equal amounts of protein from each sample loaded onto a gel and subjected to SDS-PAGE. After transferring the proteins to PVDF, the membrane is incubated with an anti-PARP antibody (Cell Signaling, 46D11), followed by stripping and re-incubation with an anti-β-Actin antibody (Invitrogen, 15G5A11/E2) for loading control. The detection was performed using an ECL reagent and visualized with a Biorad imager.
(TIF)

S5 Fig Morphologic changes in OVCAR3 and ES2 cells upon treatment with RA475. Human ovarian cancer-derived OVCAR3 or ES2 cells were seeded at 200,000/well in a 6 well format and the next day were treated with the indicated concentrations of RA475 or vehicle (DMSO) alone for 48 h. The cells were imaged by phase contrast light microscopy after 24h and 48h treatment.
(TIF)

S6 Fig RA475 induced apoptosis in a triple negative breast cancer cell line. MDA-MB-468 cells initiated apoptosis upon 12 h treatment with RA475 (1 or 2 μM) or ixazomib (1 μM) as measured by surface Annexin V display using flow cytometry.
(TIF)

S7 Fig Measurement of aqueous solubility. Aqueous solubility calibration curves for Ondonsetron (A) Up284 (B) and RA475 (C).
(TIF)

S8 Fig hERG inhibition in vitro. Thallium Flux FLIPR-Based Assay for the Identification of hERG Potassium Channel Inhibitors Assessment of compound RA475 A) Haloperidol dose-response curve. Concentration range of 0.023–50 μM (8 points, 3-fold serial dilutions) B) Dose-response curve for RA475. Concentration range of 0.045–100 μM (8 points, 3-fold serial dilutions). C) Dose-response curve for Up284. Concentration range of 0.045–100 μM (8 points, 3-fold serial dilutions).
(TIF)

S9 Fig Original gel images. (PDF)

S1 Table Cytotoxic effect of RA compounds and Up284 in various cell lines measured using the MTT assay (IC50 in μM). (DOCX)

S2 Table Stability of RA475 in murine and human liver microsomes with or without exogenous NADPH. (DOCX)

S3 Table Mouse plasma protein binding. (DOCX)

S4 Table Human plasma binding. (DOCX)

S5 Table Human serum albumin protein binding. (DOCX)

S6 Table Experimental LogD, pH 7.4. (DOCX)

S7 Table Analysis of aqueous solubility of Up284 and RA475. (DOCX)

S8 Table GSH reactivity data for reference and test compounds. (DOCX)

S9 Table Study design. (DOCX)

S10 Table Plasma concentration of RA475 in male CD1 mice following IV administration (10 mg/Kg). (DOCX)

S11 Table Plasma concentration of RA475 in male CD1 mice following IP administration (40 mg/Kg). (DOCX)

S12 Table Plasma concentration of RA475 in male CD1 mice following PO administration (40 mg/Kg). (DOCX)

S13 Table Comparison of selected PK parameters for RA475 in male CD1 mice following various dosing regimen. (DOCX)

S1 File Description of chemical synthesis of compounds. (DOCX)
