# Supplementary figures and images for: Preclinical studies of RA475, a guanidine-substituted spirocyclic candidate RPN13/ADRM1 inhibitor for treatment of ovarian cancer
Source: PLoS One. 2024 Jul 11;19(7):e0305710. doi: 10.1371/journal.pone.0305710 (PMC11239005; doi:10.1371/journal.pone.0305710)

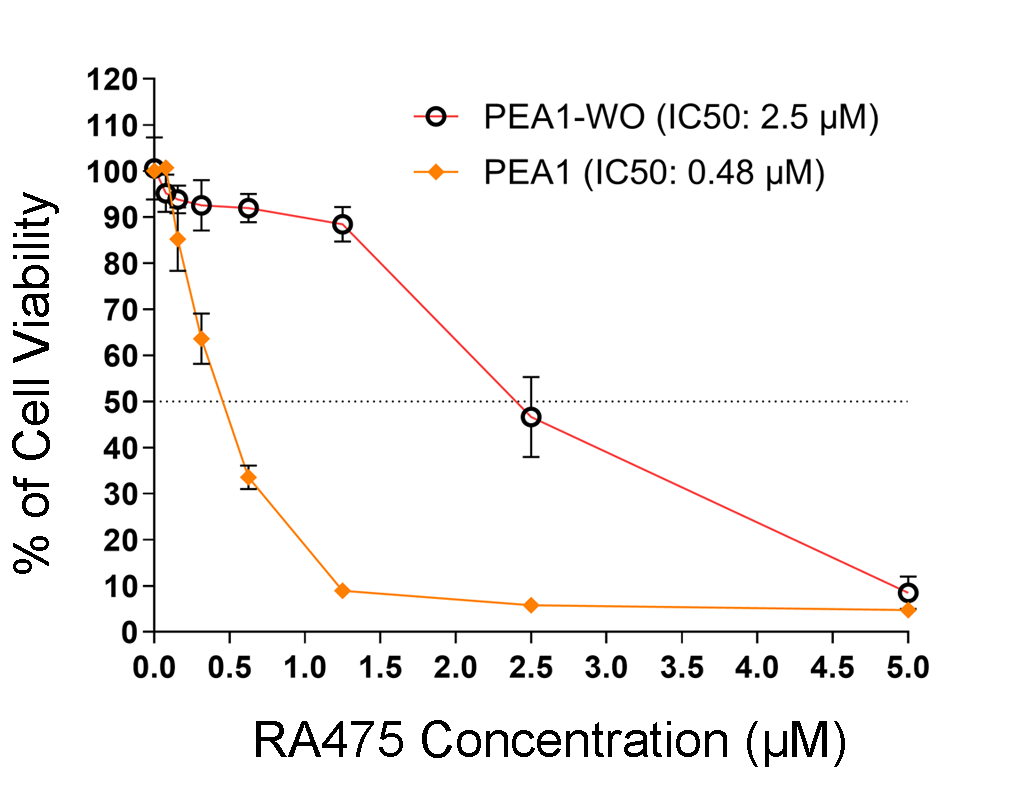

Supplement: S1 Fig — PEA1 cells were treated with RA475 for 72 h (PEA1) and in another set PEA1 cells were treated with RA475 for 1 h, and then RA475 was washed out (WO) by replacing the medium with fresh medium and cultured for an additional 71 h (PEA1-WO). Cell viability was measured using MTT assay and the data was plotted percent of control. (TIF) [file pone.0305710.s001.tif]

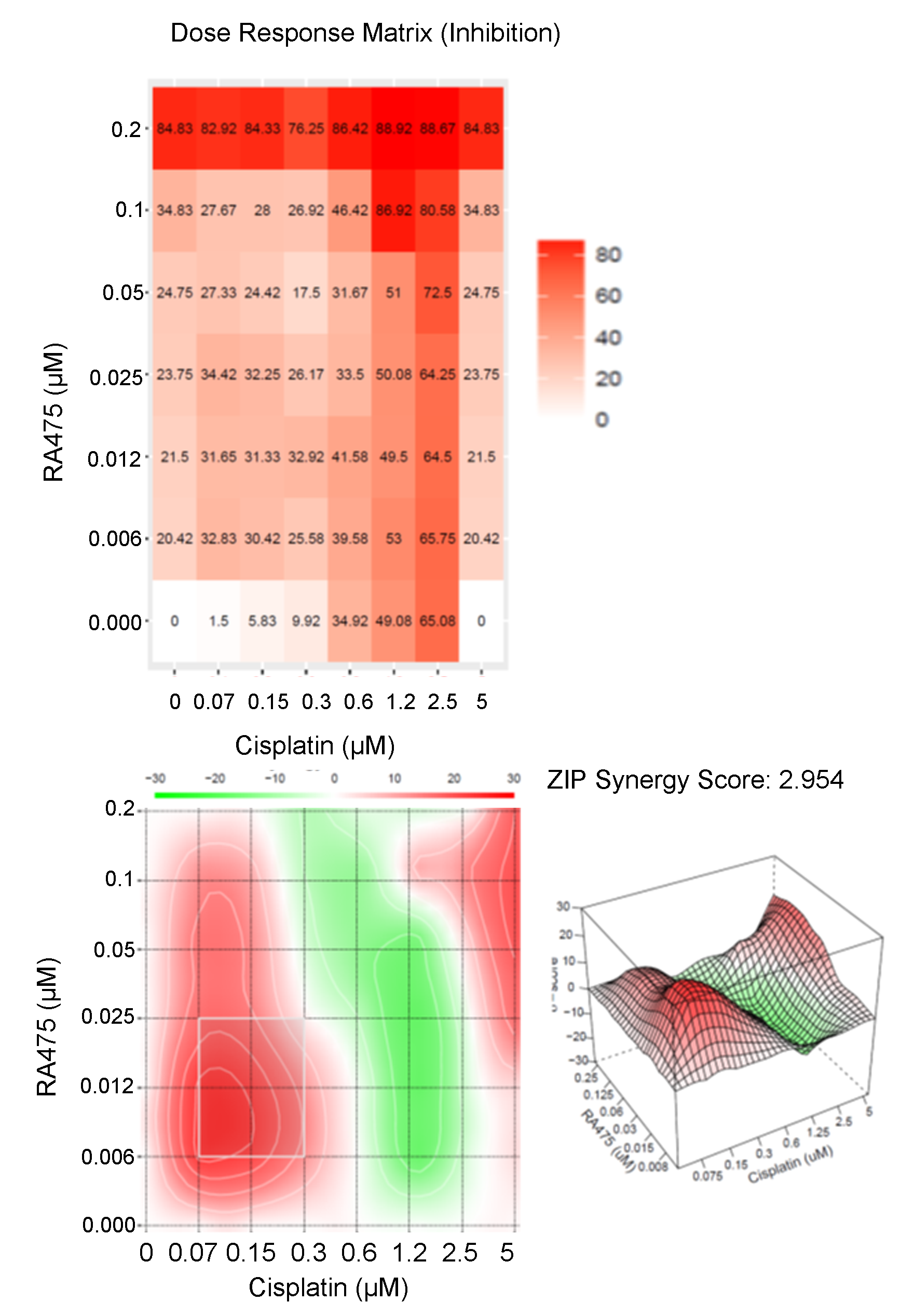

Supplement: S2 Fig — Human ovarian cancer-derived PEA1 cells in a 96 well format were treated with combinations of RA475 and cisplatin in a checker board assay format. The cells were then incubated for 72 h and cell viability was measured using MTT assay. Data were analyzed and plotted using the Synergy Finder web application. RA475 demonstrated mild synergy with cisplatin for cytotoxicity for PEA1 cells. (TIF) [file pone.0305710.s002.tif]

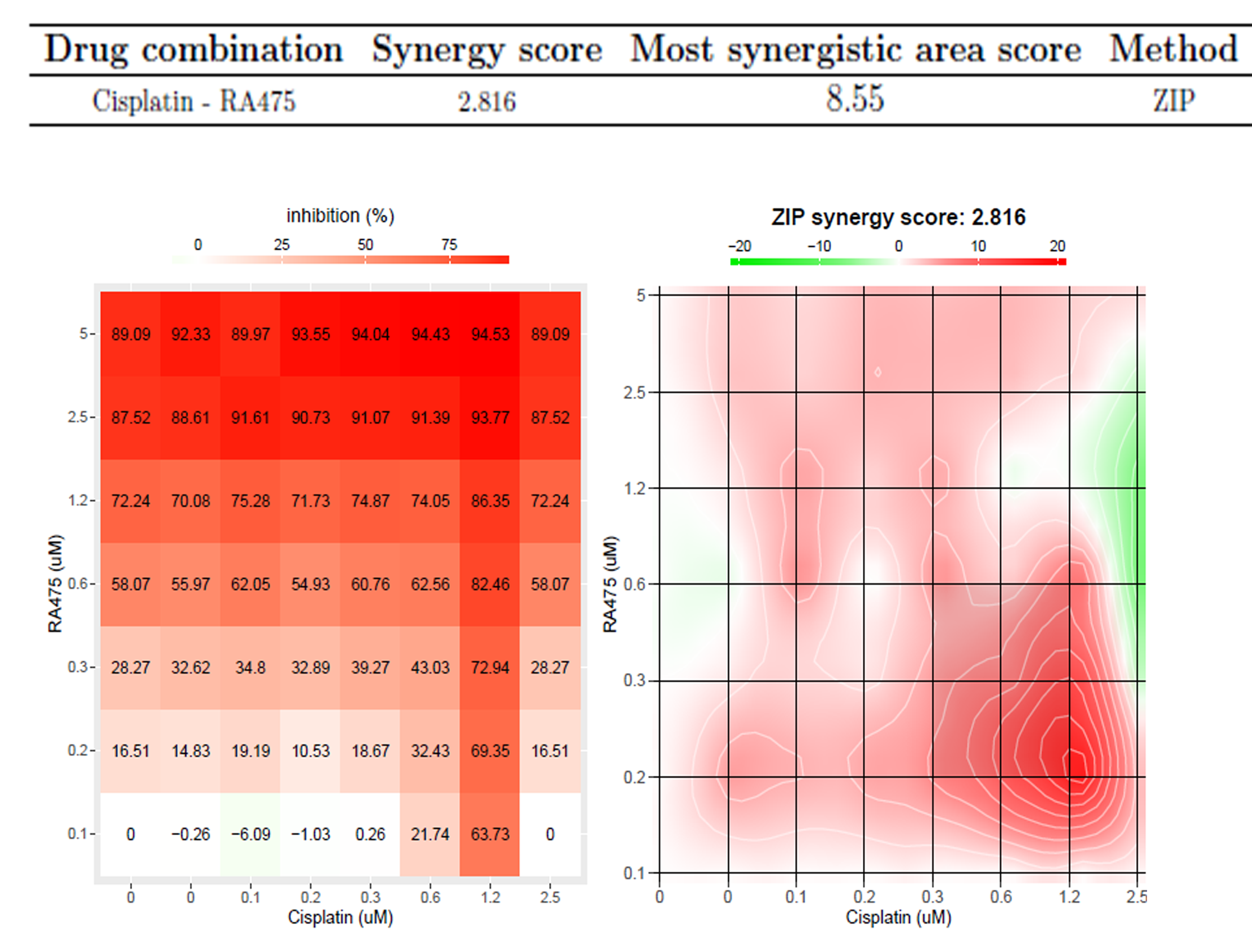

Supplement: S3 Fig — Human ovarian cancer-derived OVCAR3 cells in a 96 well format were treated with combinations of RA475 and cisplatin in a checker board assay format. The cells were then incubated for 72 h and cell viability was measured using MTT assay. Data were analyzed and plotted using the Synergy Finder web application. RA475 demonstrated mild synergy with cisplatin for cytotoxicity for OVCAR3 cells. (TIF) [file pone.0305710.s003.tif]

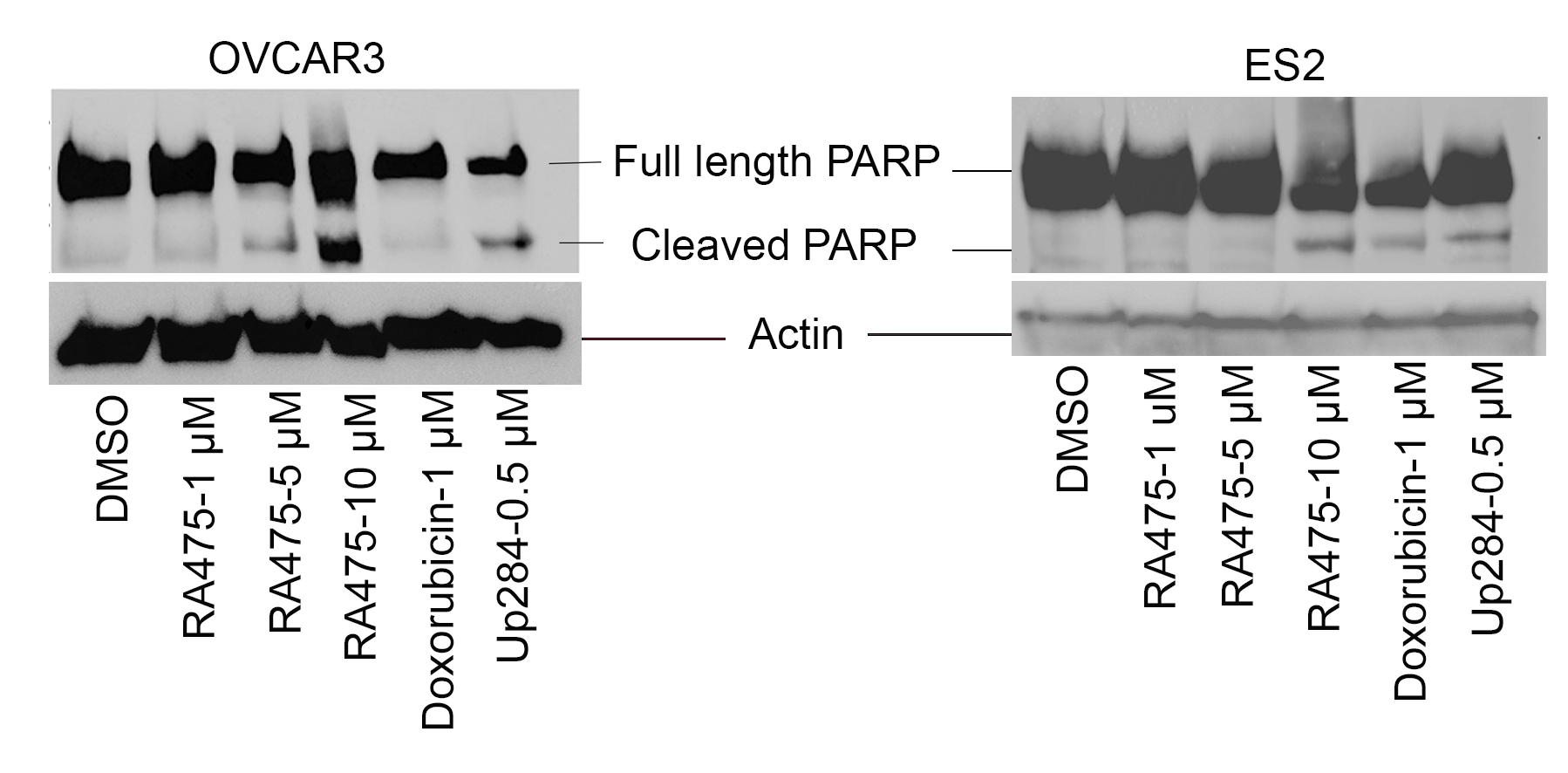

Supplement: S4 Fig — Human ovarian cancer-derived OVCAR3 or ES2 cells were seeded in a 10 cm dish at a density of 750,000 cells in 10 mL of RPMI growth medium. The following day, the cells are treated with compounds at the specified doses for 48 hours. The medium was then aspirated, and the cells are trypsinized, pelleted, washed with PBS, and pelleted again using a centrifuge. The cell pellet is lysed with 200 μL of RIPA buffer containing 2 μL of protease inhibitors at 4°C and sonicated (Misonix, 50 Amplitude) for 20 seconds in three cycles at 4°C. The lysates were centrifuged at high speed for 2 minutes to remove cell debris, and equal amounts of protein from each sample loaded onto a gel and subjected to SDS-PAGE. After transferring the proteins to PVDF, the membrane is incubated with an anti-PARP antibody (Cell Signaling, 46D11), followed by stripping and re-incubation with an anti-β-Actin antibody (Invitrogen, 15G5A11/E2) for loading control. The detection was performed using an ECL reagent and visualized with a Biorad imager. (TIF) [file pone.0305710.s004.tif]

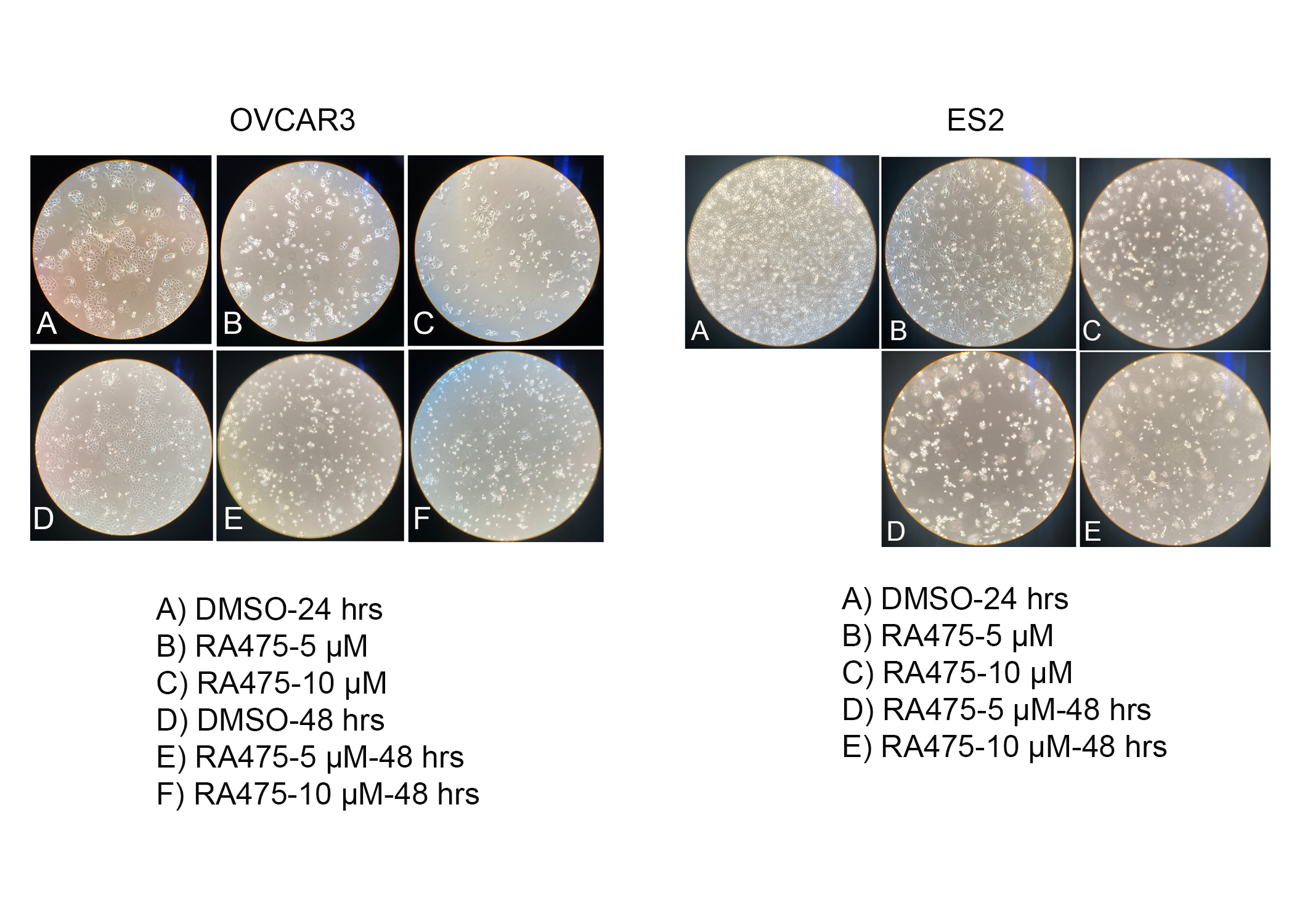

Supplement: S5 Fig — Human ovarian cancer-derived OVCAR3 or ES2 cells were seeded at 200,000/well in a 6 well format and the next day were treated with the indicated concentrations of RA475 or vehicle (DMSO) alone for 48 h. The cells were imaged by phase contrast light microscopy after 24h and 48h treatment. (TIF) [file pone.0305710.s005.tif]

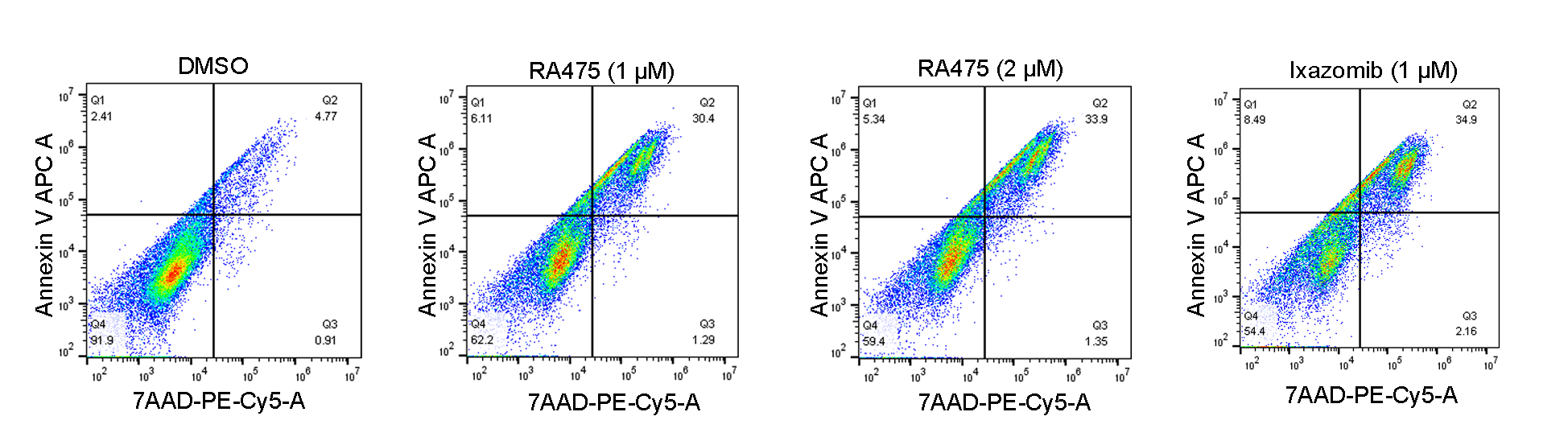

Supplement: S6 Fig — MDA-MB-468 cells initiated apoptosis upon 12 h treatment with RA475 (1 or 2 μM) or ixazomib (1 μM) as measured by surface Annexin V display using flow cytometry. (TIF) [file pone.0305710.s006.tif]

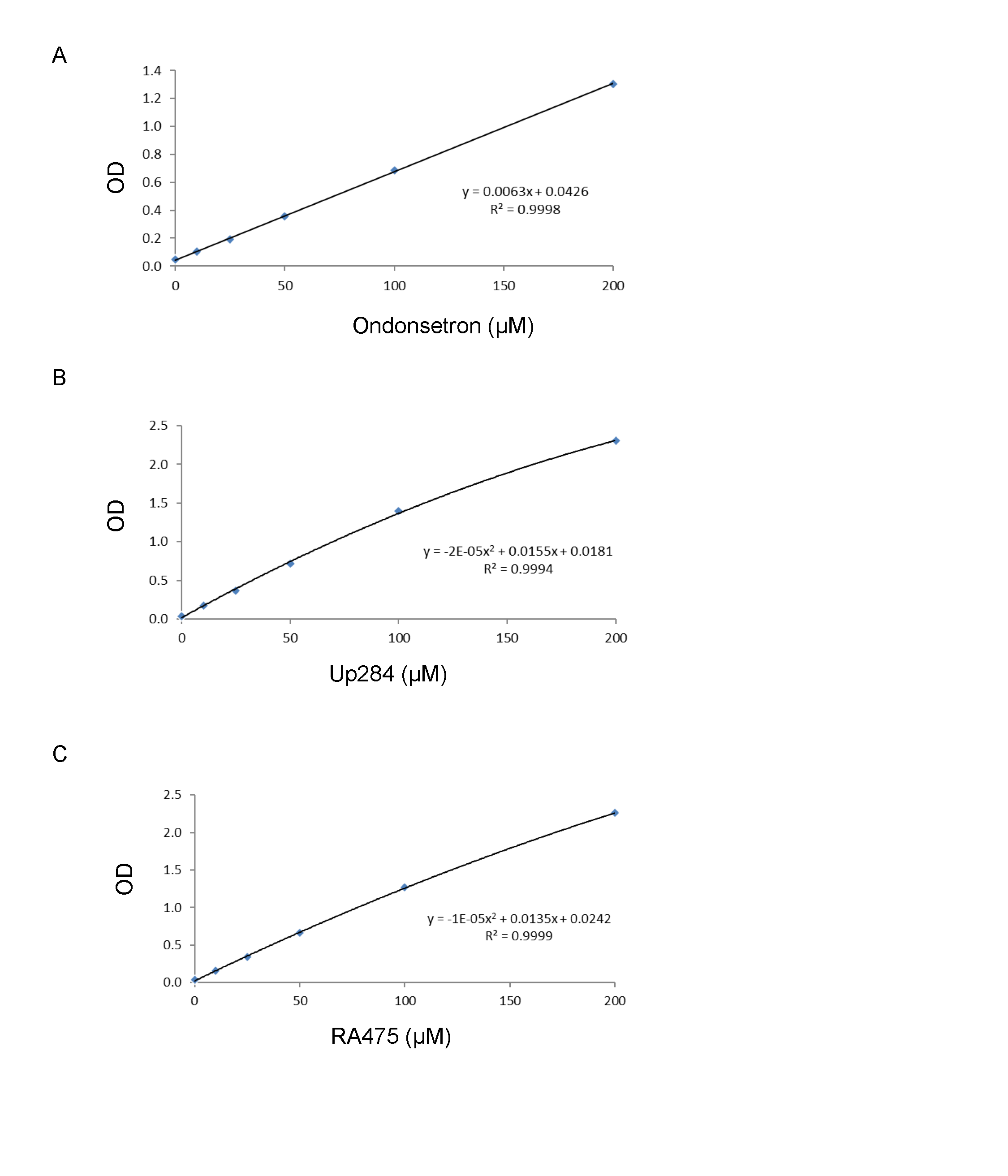

Supplement: S7 Fig — Aqueous solubility calibration curves for Ondonsetron (A) Up284 (B) and RA475 (C). (TIF) [file pone.0305710.s007.tif]

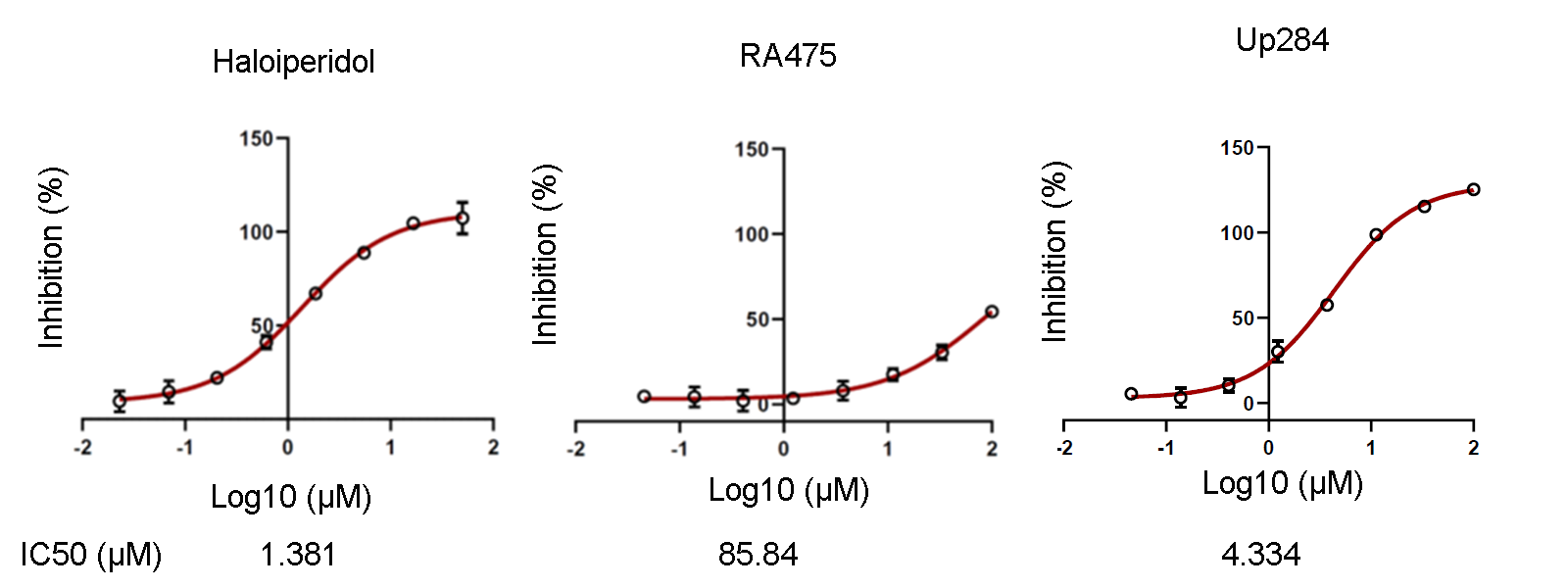

Supplement: S8 Fig — Thallium Flux FLIPR-Based Assay for the Identification of hERG Potassium Channel Inhibitors Assessment of compound RA475 A) Haloperidol dose-response curve. Concentration range of 0.023–50 μM (8 points, 3-fold serial dilutions) B) Dose-response curve for RA475. Concentration range of 0.045–100 μM (8 points, 3-fold serial dilutions). C) Dose-response curve for Up284. Concentration range of 0.045–100 μM (8 points, 3-fold serial dilutions). (TIF) [file pone.0305710.s008.tif]

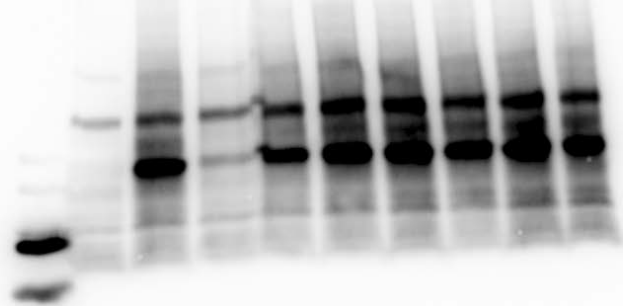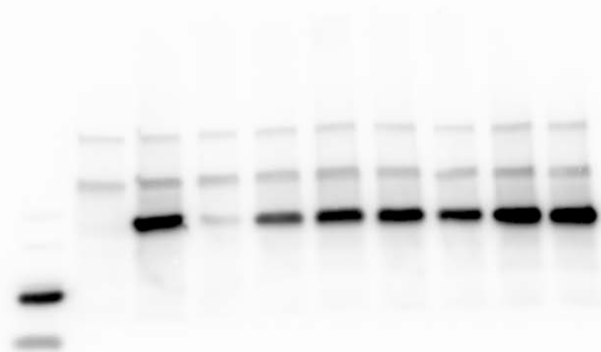

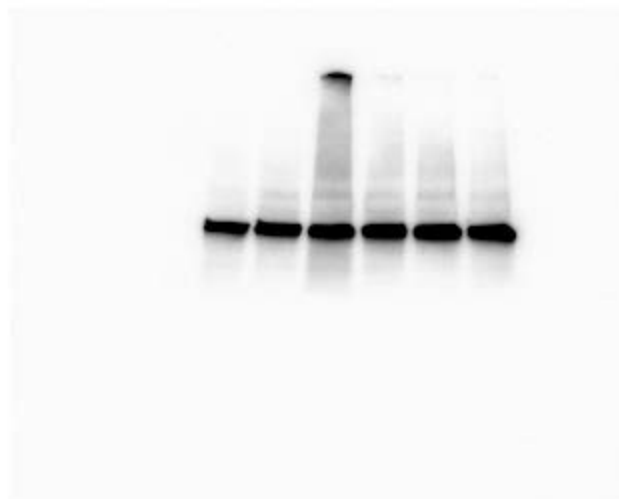

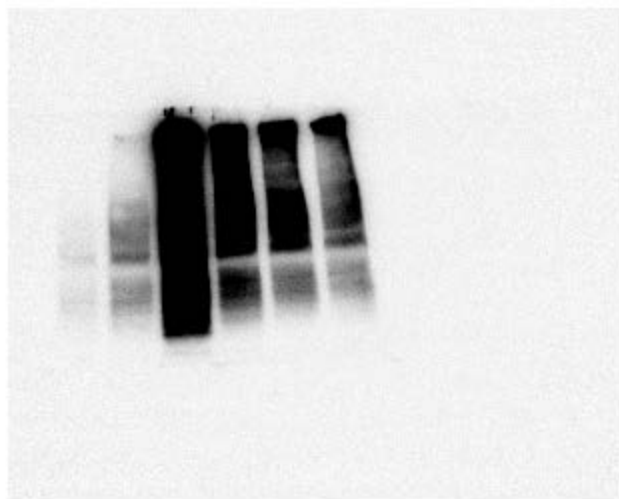

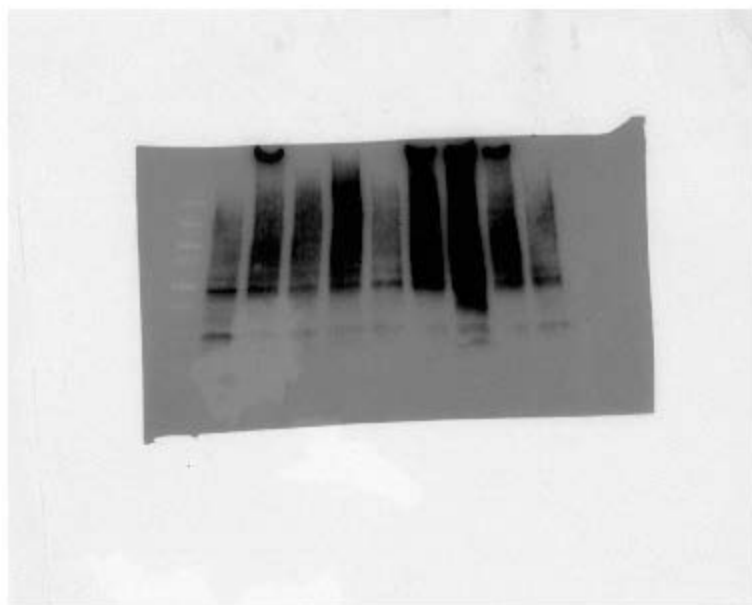

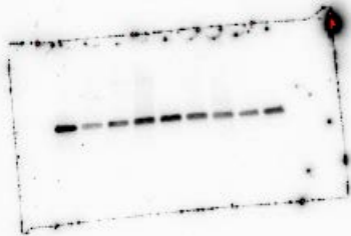

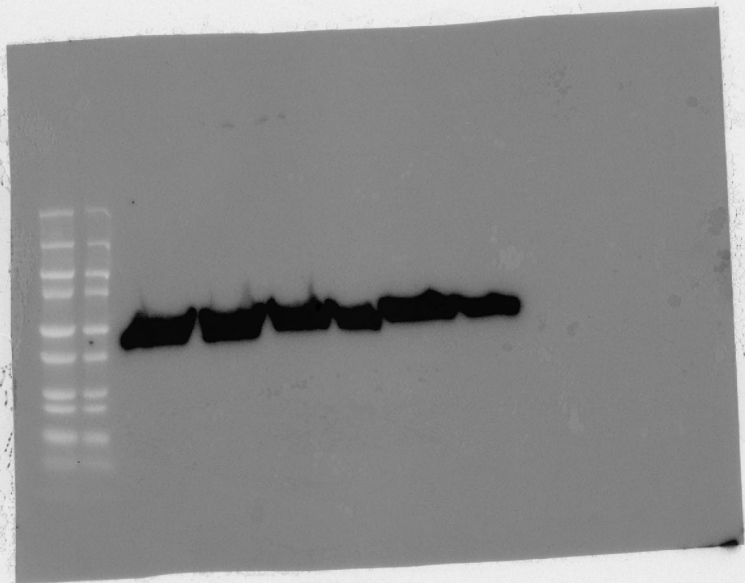

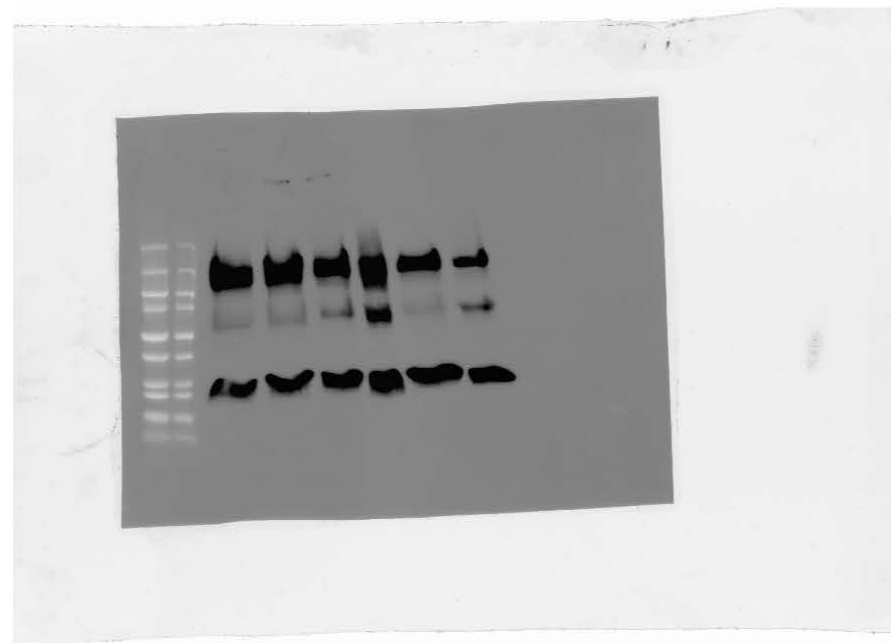

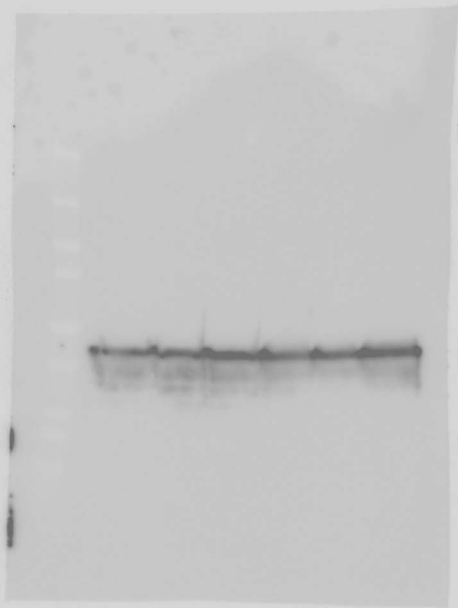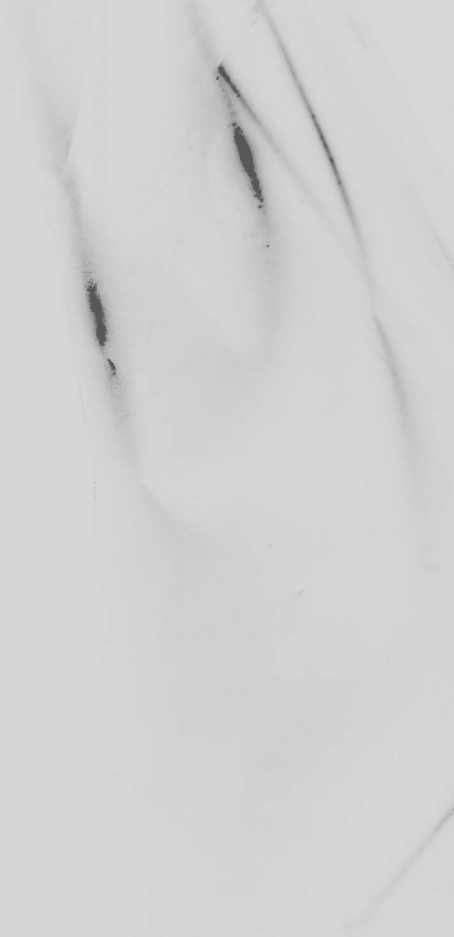

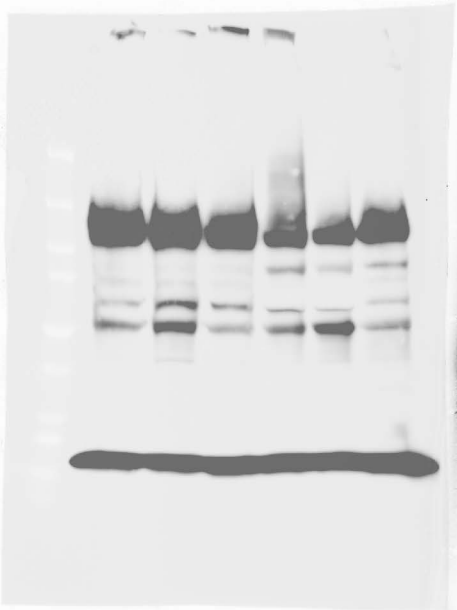

Supplement: S9 Fig — (PDF) [file pone.0305710.s009.pdf]
