# Supplementary material for: Preclinical studies of RA475, a guanidine-substituted spirocyclic candidate RPN13/ADRM1 inhibitor for treatment of ovarian cancer
Source: PLoS One. 2024 Jul 11;19(7):e0305710. doi: 10.1371/journal.pone.0305710 (PMC11239005; doi:10.1371/journal.pone.0305710)
Supplement: S1 Table — (DOCX) [file pone.0305710.s010.docx]

**Table S1:** Cytotoxic effect of RA compounds and Up284 in various cell lines measured using the MTT assay (IC50 in µM). Structures, synthesis and validation of RA475, RA477, RA479, RA482 and RA484 are presented in Supplemental Methods.

A.

| **Cell Line** | **Up284** | **RA475** | **RA477** | **RA479** | **RA482** | **RA484** |
| --- | --- | --- | --- | --- | --- | --- |
| PEA1 | 0.219 | 0.406 | 0.331 | 0.35 | 0.396 | 0.479 |
| PEA2 | 0.144 | 0.356 | 0.312 | 0.32 | 0.41 | 0.522 |

B.

| **Cell Line** | **RA475 (µM)** | **Up284 (µM)** |
| --- | --- | --- |
| TC1 | 0.217 | 0.14 |
| TSH1 | 0.114 | 0.07 |
| Hep3B | 0.997 | 0.16 |
| PEA1 | 0.406 | 0.219 |
| ID8VegF | 1.824 | 0.2 |
| LaPC4 | 2.031 | 0.51 |
| HCC1806 | 0.113 | 0.14 |
| OVCAR3 | 0.19 | 0.05 |
| SKOV3 | 0.294 | 0.071 |
| DLD1 | 0.495 | 0.11 |
| MDA-MB-468 | 0.292 | 0.072 |
| PEO23 | 0.297 | 0.112 |
| SUM149 | 0.10 | 0.09 |
| HCC1395 | 0.464 | 0.12 |
| TC1-Ova | 0.15 | 0.09 |
| B6-Ova | 0.07 | 0.05 |
| PACS | 0.581 | 0.125 |
| MC38 | 0.446 | 0.06 |
| CT26 | 0.63 | 0.05 |
| SKMEL2 | 0.156 | 0.07 |
| HT29 | 0.401 | 0.043 |
| 4T1 | 0.261 | 0.12 |
| PEO4 | 0.26 | 0.061 |
| PEA2 | 0.356 | 0.144 |
| A549 | 0.219 | 0.09 |
| Calu-1 | 0.241 | 0.065 |
| ES2 | 0.16 | 0.125 |
| HeLa | 0.213 | 0.04 |
| BT549 | 0.229 | 0.07 |
| MCF7 | 0.742 | 0.69 |
| HepG2 | 0.241 | 0.09 |
| HCT116 | 0.43 | 0.11 |
| HFF | >2.5 | >2.5 |
| Mouse keratinocytes | >2.5 | >2.5 |
