# Supplementary material for: Preclinical studies of RA475, a guanidine-substituted spirocyclic candidate RPN13/ADRM1 inhibitor for treatment of ovarian cancer
Source: PLoS One. 2024 Jul 11;19(7):e0305710. doi: 10.1371/journal.pone.0305710 (PMC11239005; doi:10.1371/journal.pone.0305710)
Supplement: S2 Table — (DOCX) [file pone.0305710.s011.docx]

**Table S2. Stability of RA475 in murine and human liver microsomes with or without exogenous NADPH**
