# Supplementary material for: Preclinical studies of RA475, a guanidine-substituted spirocyclic candidate RPN13/ADRM1 inhibitor for treatment of ovarian cancer
Source: PLoS One. 2024 Jul 11;19(7):e0305710. doi: 10.1371/journal.pone.0305710 (PMC11239005; doi:10.1371/journal.pone.0305710)
Supplement: S6 Table — (DOCX) [file pone.0305710.s015.docx]

**Table S6. Experimental LogD, pH 7.4**

| **Compound ID** | **Injection** | **S*P*** | **S*O*** | **D** | **LogD, pH 7.4** | |
| --- | --- | --- | --- | --- | --- | --- |
| Mebendazole | 1 | 8.64E+04 | 7.44E+06 | 8.61E+02 | 2.936 | **2.95** |
|  | 2 | 1.43E+05 | 1.28E+07 | 8.97E+02 | 2.953 |  |
|  | 3 | 1.48E+05 | 1.31E+07 | 8.85E+02 | 2.947 |  |
| **Up284** | 1 | 5.93E+04 | 6.81E+05 | 1.15E+02 | 2.061 | **2.12** |
|  | 2 | 5.61E+04 | 7.39E+05 | 1.32E+02 | 2.12 |  |
|  | 3 | 4.91E+04 | 7.05E+05 | 1.44E+02 | 2.158 |  |
| **RA475** | 1 | 2.75E+05 | 8.70E+05 | 3.16E+01 | 1.5 | **1.58** |
|  | 2 | 2.44E+05 | 9.38E+05 | 3.84E+01 | 1.585 |  |
|  | 3 | 2.17E+05 | 9.28E+05 | 4.27E+01 | 1.631 |  |

*Reliable measurable range is approximately -1 to 4.5
