# Supplementary material for: Preclinical studies of RA475, a guanidine-substituted spirocyclic candidate RPN13/ADRM1 inhibitor for treatment of ovarian cancer
Source: PLoS One. 2024 Jul 11;19(7):e0305710. doi: 10.1371/journal.pone.0305710 (PMC11239005; doi:10.1371/journal.pone.0305710)
Supplement: S7 Table — (DOCX) [file pone.0305710.s016.docx]

**Table S7. Analysis of aqueous solubility of Up284 and RA475**

| **Compound ID** | **PBS solubility, pH 7.4, µM** | | | **SE** |
| --- | --- | --- | --- | --- |
|  | **Incubation 1** | **Incubation 2** | **Mean** |  |
| Ondansetron | 113 | 113 | **113*** | 0.3 |
| **Up284** | 37 | 37 | **37** | 0.1 |
| **RA475** | 136 | 138 | **137** | 1.1 |

*Ondansetron solubility data are consistent with those previously obtained.
