# Supplementary material for: Preclinical studies of RA475, a guanidine-substituted spirocyclic candidate RPN13/ADRM1 inhibitor for treatment of ovarian cancer
Source: PLoS One. 2024 Jul 11;19(7):e0305710. doi: 10.1371/journal.pone.0305710 (PMC11239005; doi:10.1371/journal.pone.0305710)
Supplement: S8 Table — (DOCX) [file pone.0305710.s017.docx]

**Table S8. GSH reactivity data for reference and test compounds**

| **Compound ID** | **Time, h** | **Average Area Ratio** | | | **R2** | | **% Remaining** | | | **t1/2, h** | | | |
| --- | --- | --- | --- | --- | --- | --- | --- | --- | --- | --- | --- | --- | --- |
|  |  | **with GSH** | **without GSH** | **Normalized** | **with GSH,**  **normalized** | | **with GSH,**  **normalized** | **w/o GSH** | | **with GSH,**  **normalized** | | | **w/o GSH** |
| PCM- 0102244 | 0 | 1.00E+02 | 1.00E+02 | 9.15E+01 | 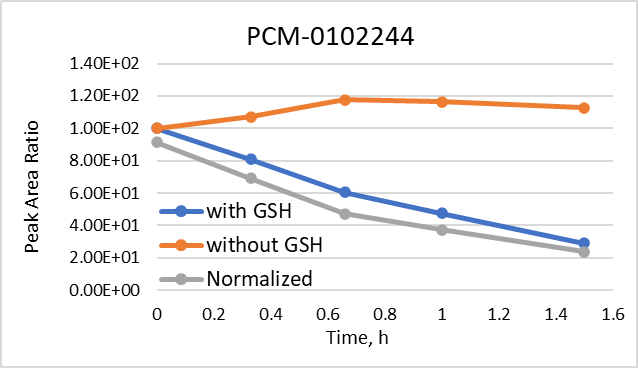 | | | | | | | | |
|  | 0.33 | 8.08E+01 | 1.07E+02 | 6.90E+01 |  |  |  |  |  |  |  |  |  |
|  | 0.66 | 6.06E+01 | 1.18E+02 | 4.71E+01 |  |  |  |  |  |  |  |  |  |
|  | 1 | 4.74E+01 | 1.16E+02 | 3.73E+01 |  |  |  |  |  |  |  |  |  |
|  | 1.5 | 2.90E+01 | 1.13E+02 | 2.35E+01 | 0.9962 | | 24 | 113 | | 0.765 | | | >6 |
| RA475 | 0 | 1.00E+02 | 1.00E+02 | 9.10E+01 | 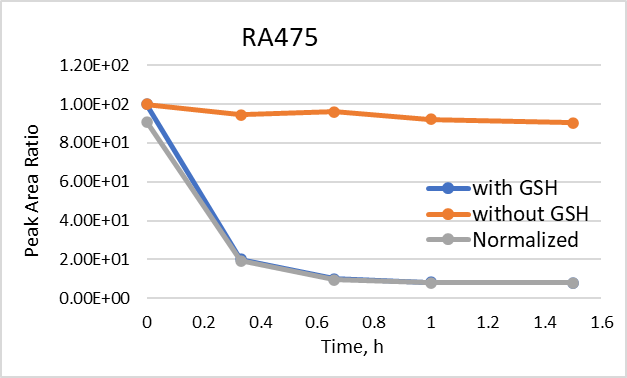 | | | | | | | | |
|  | 0.33 | 2.00E+01 | 9.44E+01 | 1.93E+01 |  |  |  |  |  |  |  |  |  |
|  | 0.66 | 1.02E+01 | 9.60E+01 | 9.62E+00 |  |  |  |  |  |  |  |  |  |
|  | 1 | 8.18E+00 | 9.22E+01 | 8.07E+00 |  |  |  |  |  |  |  |  |  |
|  | 1.5 | 7.92E+00 | 9.03E+01 | 7.98E+00 | 0.9536 | | 8 | 90 | | 0.204 | | | >6 |
| Up284 | 0 | 1.00E+02 | 1.00E+02 | 8.68E+01 | 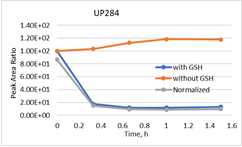 | | | | | | | | |
|  | 0.33 | 1.76E+01 | 1.03E+02 | 1.48E+01 |  |  |  |  |  |  |  |  |  |
|  | 0.66 | 1.18E+01 | 1.13E+02 | 9.11E+00 |  |  |  |  |  |  |  |  |  |
|  | 1 | 1.20E+01 | 1.18E+02 | 8.78E+00 |  |  |  |  |  |  |  |  |  |
|  | 1.5 | 1.30E+01 | 1.18E+02 | 9.58E+00 | 0.9019 | 10 | | | 118 | | 0.203 | >6 | |
