# Supplementary material for: Preclinical studies of RA475, a guanidine-substituted spirocyclic candidate RPN13/ADRM1 inhibitor for treatment of ovarian cancer
Source: PLoS One. 2024 Jul 11;19(7):e0305710. doi: 10.1371/journal.pone.0305710 (PMC11239005; doi:10.1371/journal.pone.0305710)
Supplement: S9 Table — (DOCX) [file pone.0305710.s018.docx]

**Table S9: Study Design**

| Number of Animals | Compound ID | Formulation | Delivery Route | Target Dose Level (mg/kg) | Target Dose Concentration (mg/ml) | Target Dose Volume (ml/kg) |
| --- | --- | --- | --- | --- | --- | --- |
| 28 | **RA475** | DMSO – 25% b-hydroxypropyl cyclodextrin in water (6%:94%; v/v) | IV | 10 | 2 | 5 |
| 1 | Vehicle dosed |  | IV | 0 | 0 | 5 |
| 28 | **RA475** | DMSO – 25% b-hydroxypropyl cyclodextrin in water (6%:94%; v/v) | IP | 40 | 8 | 5 |
| 1 | Vehicle dosed |  | IP | 0 | 0 | 5 |
| 28 | **RA475** | DMSO – 25% b-hydroxypropyl cyclodextrin in water (6%:94%; v/v) | PO | 40 | 8 | 5 |
| 1 | Vehicle dosed |  | PO | 0 | 0 | 5 |
