# Supplementary material for: Preclinical studies of RA475, a guanidine-substituted spirocyclic candidate RPN13/ADRM1 inhibitor for treatment of ovarian cancer
Source: PLoS One. 2024 Jul 11;19(7):e0305710. doi: 10.1371/journal.pone.0305710 (PMC11239005; doi:10.1371/journal.pone.0305710)
Supplement: S11 Table — (DOCX) [file pone.0305710.s020.docx]

**Table S11: Plasma concentration of RA475 in male CD1 mice following IP administration (40 mg/Kg)**

| **Sample collection**  **time point, min** | **Plasma concentration (ng/ml)** | | | | | | |
| --- | --- | --- | --- | --- | --- | --- | --- |
|  | **Group A** | **Group B** | **Group C** | **Group D** | **Mean** | **SD** | **SE** |
| 0 | BQL |  |  |  | **BQL** | ND | ND |
| 15 | 11740 | 11770 | 6685 | 15900 | **11524** | 3771 | 1886 |
| 30 | 8666 | 168 | 9718 | 18830 | **9346** | 7633 | 3816 |
| 60 | 4552 | 3825 | 208 | 5740 | **3581** | 2383 | 1192 |
| 120 | 1453 | 996 | 282 | 1144 | **969** | 496 | 248 |
| 240 | 356 | 484 | 577 | 614 | **508** | 115 | 58 |
| 360 | 327 | 174 | BQL | BQL | **125** | 158 | 79 |
| 480 | 105 | 118 | 174 | 125 | **131** | 30 | 15 |
