# Supplementary material for: Preclinical studies of RA475, a guanidine-substituted spirocyclic candidate RPN13/ADRM1 inhibitor for treatment of ovarian cancer
Source: PLoS One. 2024 Jul 11;19(7):e0305710. doi: 10.1371/journal.pone.0305710 (PMC11239005; doi:10.1371/journal.pone.0305710)
Supplement: S12 Table — (DOCX) [file pone.0305710.s021.docx]

**Table S12: Plasma concentration of RA475 in male CD1 mice following PO administration (40 mg/Kg)**

| **Sample collection**  **time point, min** | **Plasma concentration (ng/ml)** | | | | | | |
| --- | --- | --- | --- | --- | --- | --- | --- |
|  | **Group A** | **Group B** | **Group C** | **Group D** | **Mean** | **SD** | **SE** |
| 0 | BQL |  |  |  | **BQL** | ND | ND |
| 15 | 156 | 211 | 126 | 73 | **141** | 58 | 29 |
| 30 | 69 | 60 | 57 | 52 | **60** | 7 | 4 |
| 60 | 29 | 33 | 35 | 44 | **35** | 7 | 3 |
| 120 | 20 | 29 | 19 | 11 | **20** | 7 | 4 |
| 240 | BQL | 20* | BQL | BQL | **BQL** | ND | ND |
| 360 | BQL | BQL | BQL | BQL | **BQL** | ND | ND |
| 480 | BQL | BQL | BQL | BQL | **BQL** | ND | ND |
