# Supplementary material for: Preclinical studies of RA475, a guanidine-substituted spirocyclic candidate RPN13/ADRM1 inhibitor for treatment of ovarian cancer
Source: PLoS One. 2024 Jul 11;19(7):e0305710. doi: 10.1371/journal.pone.0305710 (PMC11239005; doi:10.1371/journal.pone.0305710)
Supplement: S13 Table — (DOCX) [file pone.0305710.s022.docx]

**Table S13: Comparison of Selected PK parameters for RA475 in male CD1 mice following various dosing regimen**

| Sample | Administration | Dose, mg/kg | Pharmacokinetic Parameters | | | | | | | |
| --- | --- | --- | --- | --- | --- | --- | --- | --- | --- | --- |
|  |  |  | T_max_  (h) | C_max_ (ng/ml) | AUClast (ng*h/mL) | AUC∞ (ng*h/mL) | T1/2  (h) | V or V/F  (L/kg) | Cl or Cl/F  (mL/min/kg) | Bioavailability  (%) |
| Plasma | IV | 10 | 0.083 | 10504 | 2731 | 2781 | 2.1 | 10.9 | 3.6 | ND |
|  | PO | 40 | 0.25 | 142 | 118 | 139 | 1.6 | 668.1 | 288.7 | 1.1 |
|  | IP | 40 | 0.25 | 11524 | 11279 | ND | ND | ND | ND | 103.0 |
