# Supplementary material for: Preclinical studies of RA475, a guanidine-substituted spirocyclic candidate RPN13/ADRM1 inhibitor for treatment of ovarian cancer
Source: PLoS One. 2024 Jul 11;19(7):e0305710. doi: 10.1371/journal.pone.0305710 (PMC11239005; doi:10.1371/journal.pone.0305710)
Supplement: S1 File — (DOCX) [file pone.0305710.s023.docx]

**RA COMPOUND SYNTHESIS AND ANALYSIS:**

**Synthetic Procedure for RA475:** 1,3-bis((E)-4-cyanobenzylidene)-2-oxo-7-azaspiro[3.5]nonane-7-carboximidamide dihydrochloride

Commercially available A1 (1 mmol) was dissolved in Ethanol (20 mL) and 30% NaOH solution was added drop wise at 0 °C. Next, corresponding benzaldehyde (2 mmol) was added to the solution. Stirring continued for 30 min at room temperature and ethanol was removed under reduced pressure and the crude was extracted with water and ethyl acetate. Ethyl acetate layer was collected and dried over sodium sulfate and concentrated under vacuum. The crude compound was purified by column chromatography using hexanes and ethyl acetate to afford compound A2. Compound A2 was added to 4M HCl in dioxane and stirred at room temperature for 30 min. Diethyl ether was added and the yellow precipitate was collected and washed several times with diethyl ether and hexanes. Compound A3 was dried and collected. Compound A3 (1 mmol) was dissolved in acetonitrile (20 mL) and triethyl amine (6 mmol) and N,N-bis-boc-1-guanylpyrazole (1.1 mmol) was added subsequently and refluxed overnight. Solvents were removed under reduced pressure and the crude was purified by column chromatography using hexanes and ethyl acetate as solvents to afford compound A4. Compound A4 was dissolved in 4M HCl in dioxane and stirred at room temperature for 30 min. Diethyl ether was added and the yellow precipitate was collected and washed several times with diethyl ether and hexanes. Compound RA475 was dried and collected. The resulting RA475 had a purity of >95% as confirmed by NMR and LCMS.

MS: m/z: 408 (M+1).

1H NMR (400 MHz, DMSO-d6): δ 8.31 (d, J = 8.4 Hz, 4H), 7.79 (d, J = 8.4 Hz, 4H), 7.04 (s, 2H), 3.84 – 3.78 (m, 4H), 2.17 – 2.13 (m, 4H);

13C NMR (400 MHz, DMSO-d6): δ 185.9, 156.2, 154.5 (2C), 138.7 (2C), 132.4 (4C), 132.2 (2C), 130.3 (4C), 118.6 (2C), 112.1 (2C), 45.1, 42.8 (2C), 34.7 (2C)


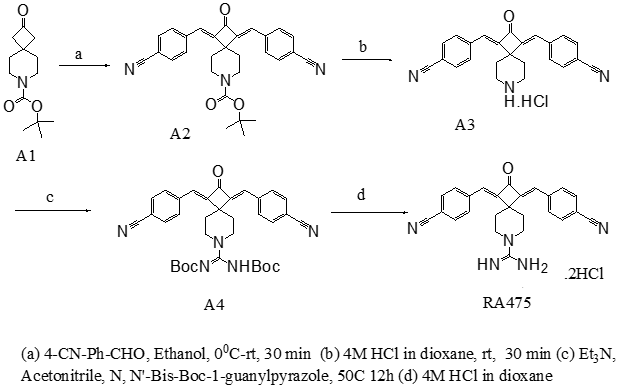

**Synthesis of RA477:** 1,3-bis((E)-4-cyanobenzylidene)-2-oxo-6-azaspiro[3.4]octane-6-carboximidamide dihydrochloride

RA477 was prepared through a comparable synthetic procedure as RA475, employing suitable starting materials (A5) and corresponding aldehyde (4-cyano benzaldehyde)

MS: m/z: 394 (M+1).

**Synthesis of RA479:** 1,3-bis((E)-4-cyano-3-fluorobenzylidene)-2-oxo-6-azaspiro[3.4]octane-6-carboximidamide dihydrochloride

RA479 was prepared through a comparable synthetic procedure as RA477, employing suitable starting materials (A5) and corresponding aldehyde (3F, 4CN- benzaldehyde)

MS: m/z: 430 (M+1).

**Synthesis of RA482:** 1,3-bis((E)-4-cyano-3-fluorobenzylidene)-2-oxo-7-azaspiro[3.5]nonane-7-carboximidamide dihydrochloride

RA482 was prepared through a comparable synthetic procedure as RA475, employing suitable starting materials (A1) and corresponding aldehyde (3F, 4CN- benzaldehyde)

MS: m/z: 444 (M+1).

**Synthesis of RA484:** 4,4'-((1E,1'E)-(7-(imino(morpholino)methyl)-2-oxo-7-azaspiro[3.5]nonane-1,3-diylidene)bis(methaneylylidene))dibenzonitrile

Compound **A3** (500 mg, 1 eq) free amine was dissolved in DCM (20 mL), treated with sat. aq. NaHCO_3_ (5 mL) and cooled to 0^0^C for 10 min. Cyanogen bromide (144 mg, 1.1 eq) was added portion wise and the resulting reaction mixture was stirred at 25^0^C for 5 h. The solvents were removed in-vacuo and the residue was basified with sat. aq. NaHCO_3_ solution. The precipitated solids were removed by filtration and washed with DCM 5 to 6 times. The combined filtrate and washings were then dried over Sodium Sulfate and the solvent was removed in-vacuo and the crude product was purified by silicagel column chromatography using ethyl acetate/hexanes to give 4,4'-((1E,1'E)-(7-cyano-2-oxo-7-azaspiro[3.5]nonane-1,3-diylidene)bis(methaneylylidene))dibenzonitrile **(A3-CN**) (276 mg, 71%) as a yellow powder.

In a nitrogen atmosphere, a 50 mL round-bottomed flask equipped with reflux condenser, was placed with A3-CN (1 eq) and morpholine (2 eq)) in DMF (5 mL) then heated at 100 ^o^C for 12h. After it cooled to room temperature, a solution of 35% NaOH was added under vigorous stirring, then extracted with Ethyl acetate (2×50 mL), dried over NaSO_4_, after removal of the solvents, the residue was purified using silca gel column chromatography to afford the RA484 (yield 22%).

MS: m/z: 478 (M+1).

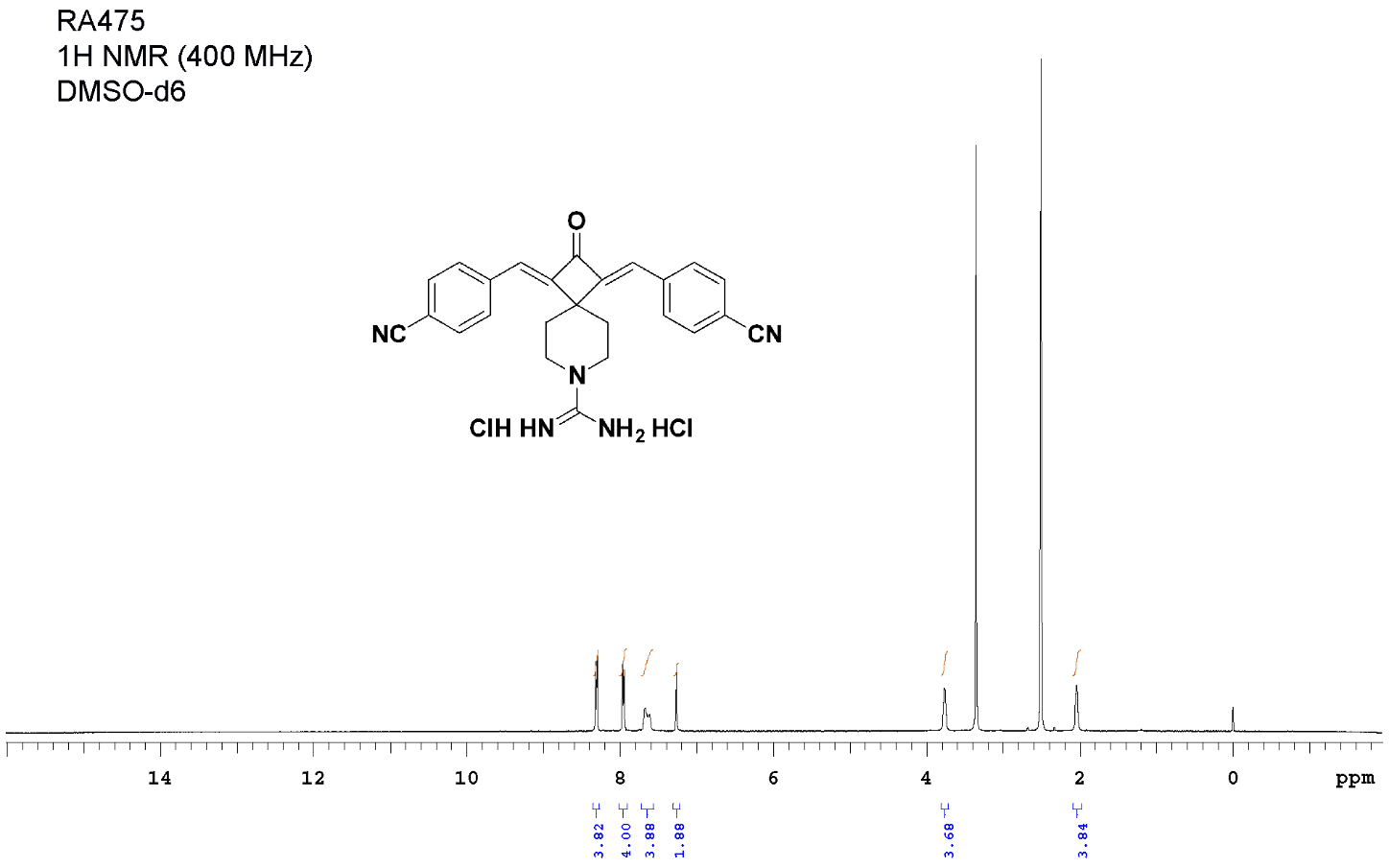


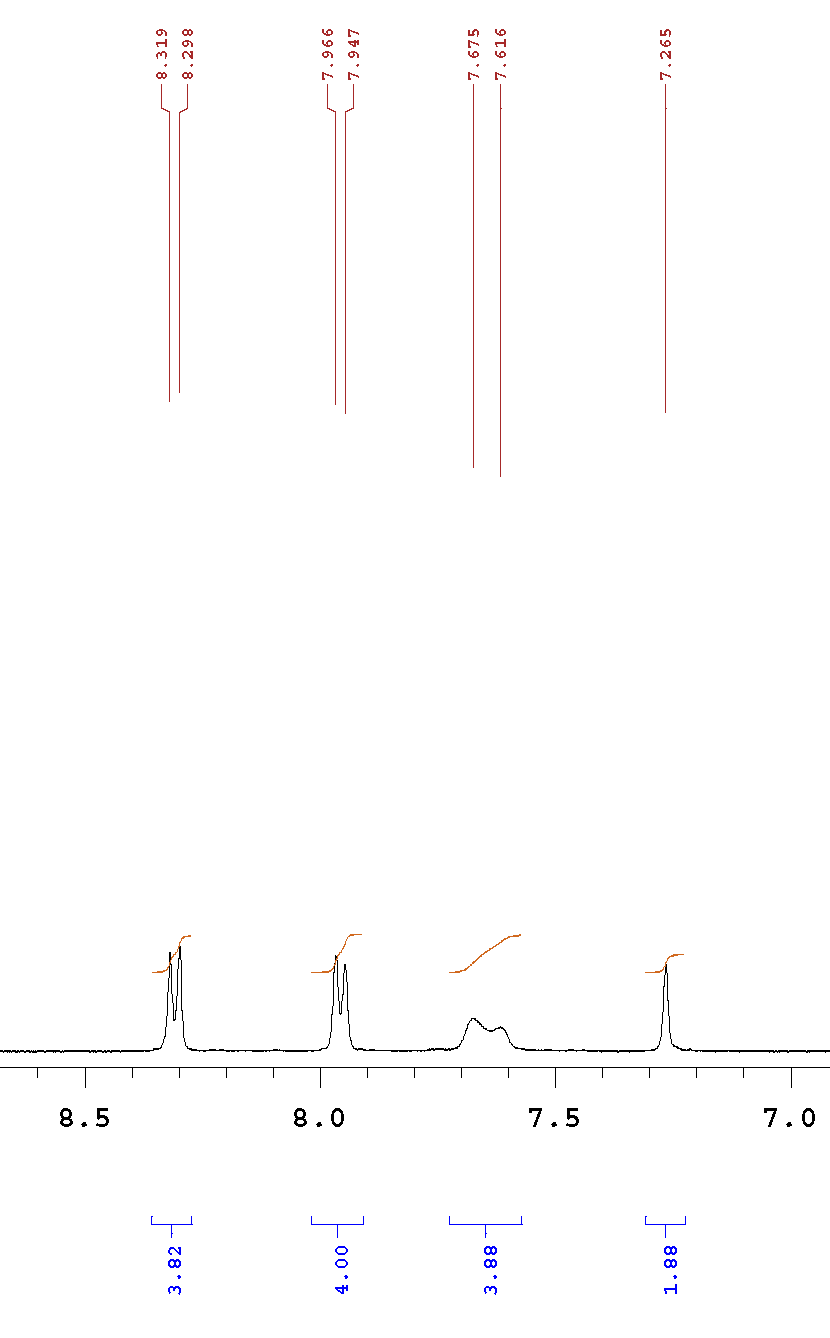


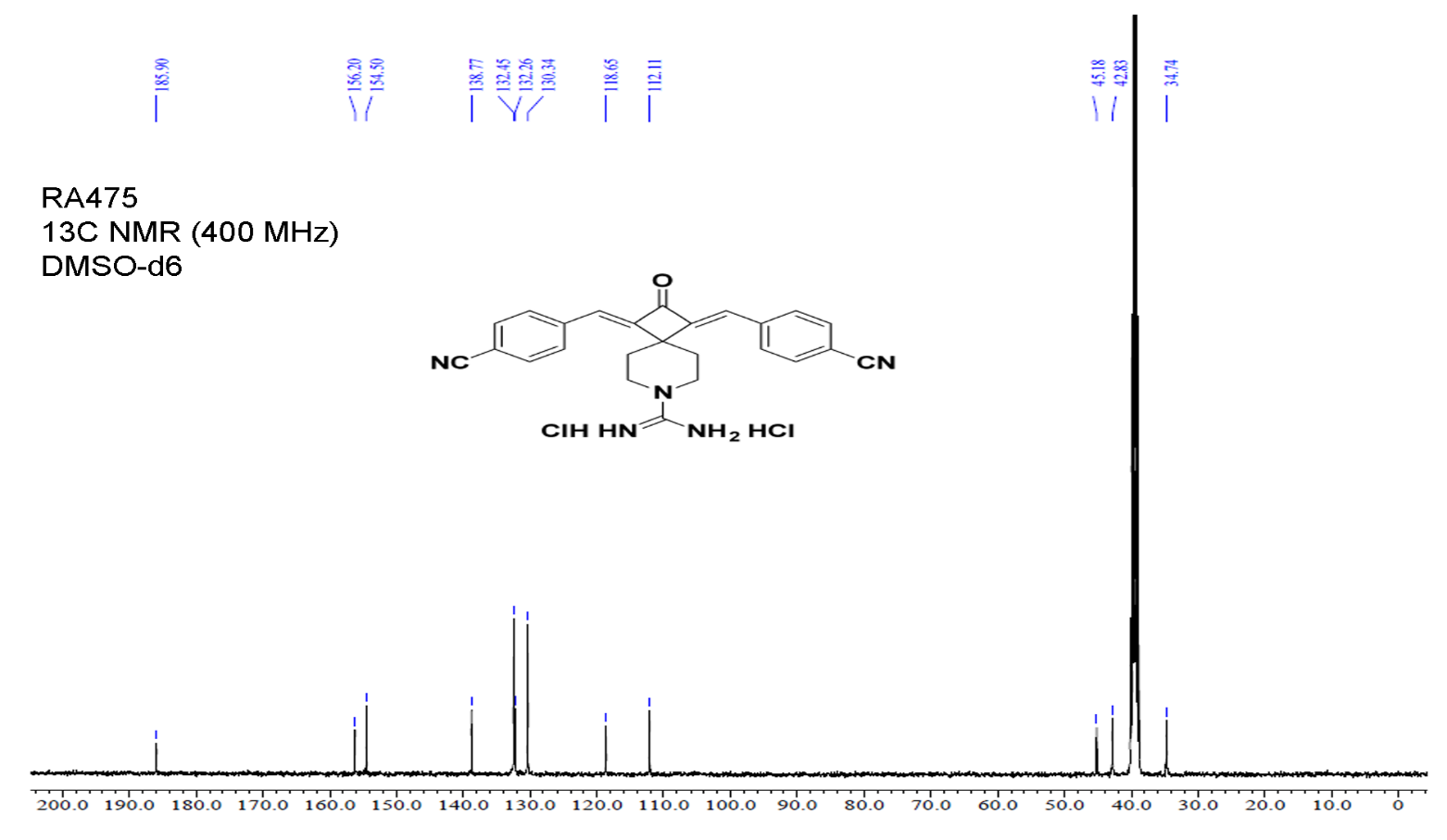

**RA475 MASS SPECTRA**

**
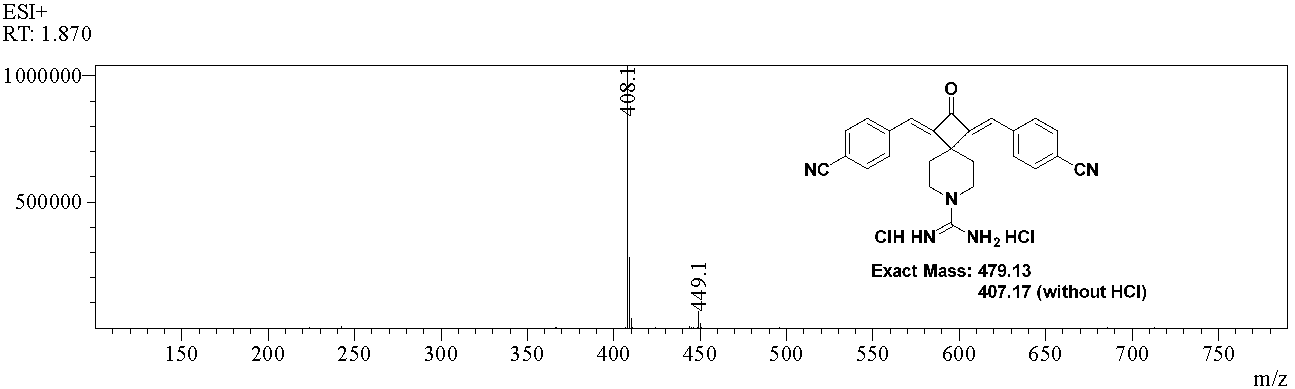
**

**RA475 HPLC**


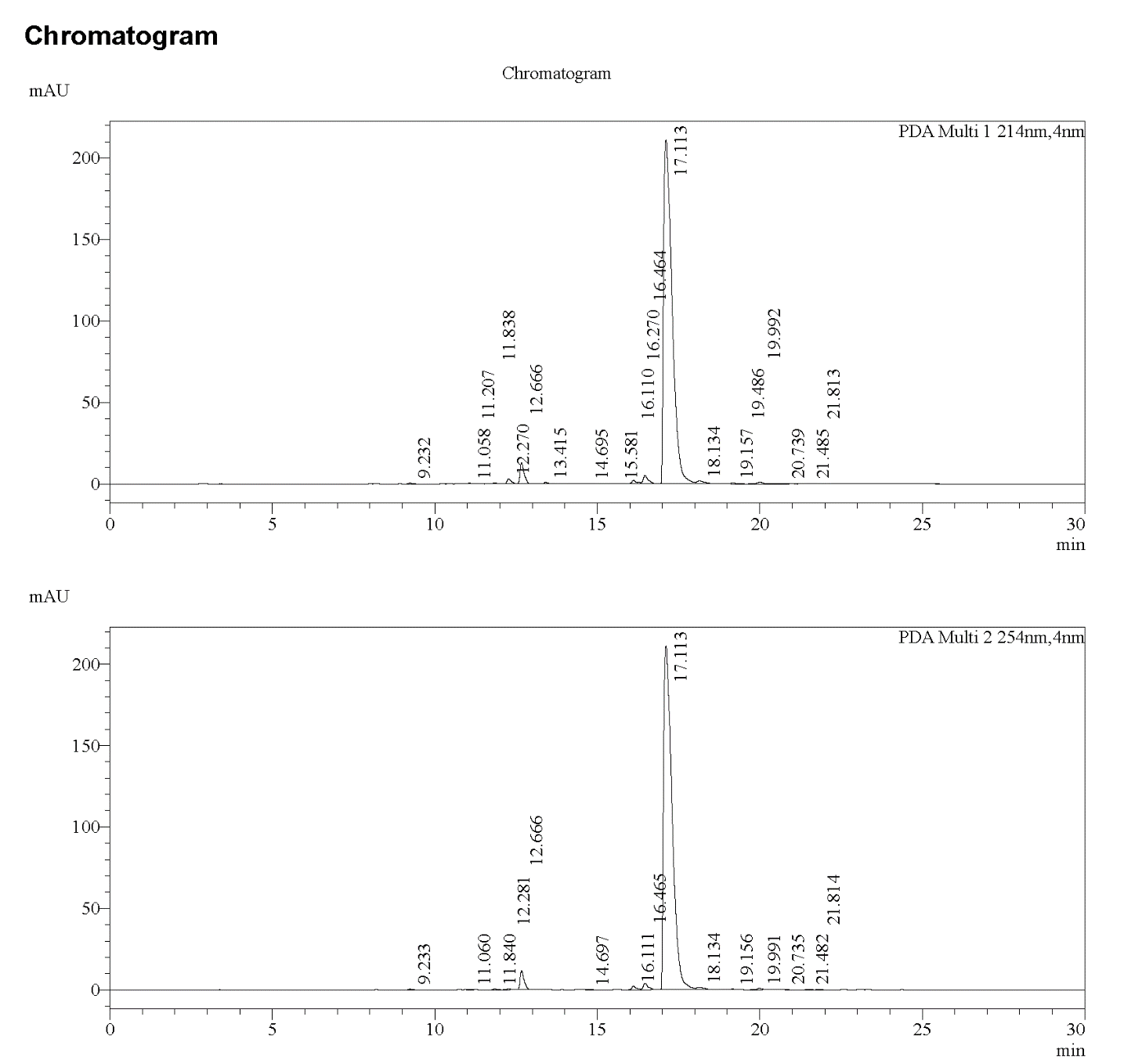


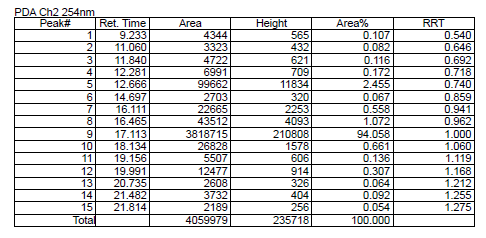


**HPLC Method Development for RA475:**

Column: Gemini, C18 5 µm 4.6*250mm

Mobile Phase:

A) Acetonitrile

B) 10 mM Ammonium Bicarbonate

Gradient T/%B:

0 min: 90% B

10 min: 50% B

20 min: 10% B

30 min: 10% B

Flow Rate: 1 mL/min

Procedure:

1. The HPLC system was set up according to the instrument manufacturer's instructions.
2. The Gemini C18 column (5 µm, 4.6*250mm) was installed in the column compartment.
3. The mobile phase was prepared by mixing Acetonitrile (Mobile Phase A) and 10 mM Ammonium Bicarbonate (Mobile Phase B) in the desired proportions.
4. The initial mobile phase composition was set to 90% B and 10% A.
5. The flow rate was set to 1 mL/min.
6. The gradient program was initiated as follows:
   - 0 min: 90% B, 10% A
   - 10 min: 50% B, 50% A
   - 20 min: 10% B, 90% A (held for 10 minutes)
   - 30 min: 10% B, 90% A (held for 10 minutes)
7. The sample was injected onto the column.
8. The elution of the compounds of interest was analyzed using appropriate detection methods such as UV or MS.
9. The chromatographic data was recorded, and the peaks were analyzed accordingly.
